# Predicted high affinity binding of prion PRPC protein to Human Leukocyte Antigen (HLA)

**Published:** 2026-03-20

**Authors:** Apostolos P. Georgopoulos, Lisa M. James, Matthew Sanders

**Affiliations:** 1 The HLA and Chronic Diseases Research Groups, Brain Sciences Center, Department of Veterans Affairs Health Care System, Minneapolis, Minnesota, USA; 2 Department of Neuroscience, University of Minnesota Medical School, Minneapolis, Minnesota, USA; 3 Institute for Health Informatics, University of Minnesota Medical School, Minneapolis, Minnesota, USA; 4 Department of Psychiatry, University of Minnesota Medical School, Minneapolis, Minnesota, USA

**Keywords:** Prion PRP^C^ protein, Human Leukocyte Antigen (HLA), Autoimmunity, Binding affinity

## Abstract

Misfolding of the cellular prion protein (PRP^C^) is associated with fatal neurodegenerative prion diseases for which no treatments are currently available. Although the immune system is generally non-responsive (tolerant) to self-proteins such as PRP^C^, evidence of anti-prion antibodies suggests escape from self-tolerance in some individuals and supports the potential for the human immune system to be leveraged against prion disease. Human leukocyte antigen (HLA) plays a central role in rejecting endogenous non-self proteins (e.g. cancer neoantigens) by activating CD8+ cytolytic T cells via the Class I system (HLA-I) and CD4+ helper T cells via the Class II (HLA-II) system. Here we investigated the predicted binding affinity of 334 HLA molecules with all possible linear 9-mer (for HLA-I) and 15-, 18- and 22-mer (for HLA-II) PRP^C^ peptides to identify peptide-HLA (pHLA) complexes with strong predicted binding (IC_50_ < 50 nM). We found that 12.4% of all prion peptides tested showed strong binding affinity to HLA molecules and that 20.2% of HLA alleles were able to bind strongly with PRP^C^ peptides. These findings suggest that carriers of certain HLA alleles that are capable of binding strongly to PRP^C^ peptides may have enhanced protection against prion disease, through reduction in the overall amount of PRP^C^ available for conversion to the misfolded, infectious scrapie isoform (PRP^Sc^) of PRP^C^ and, potentially, by destroying it. These findings have implications for other disorders including common neurodegenerative diseases characterized by protein misfolding (e.g. α-synuclein, huntingtin, amyloid, tau, etc.).

## Introduction

Prion disease is an infectious, fatal neurodegenerative disease with global prevalence and no known treatment^1–8^. It is due to the posttranslational misfolding of the cellular prion protein (PRP^C^), a common, naturally occurring protein. PrP^C^ is conserved across species and is found in numerous tissues throughout the brain and periphery^9^, including red blood cells^10^, platelets^11^ and CD8+/CD4+ T lymphocytes^12^. While PrP^C^ has been implicated in several physiological functions of the nervous and immune systems, the specific roles of PrP^C^ are unclear and possibly redundant with other proteins as evidenced by contradictory and/or null findings from PrP^C^ knock-out studies^9,14^. What is clear is that misfolding of PrP^C^ due to genetic mutations in the human major prion protein (PRNP) gene or various conditions within cells is associated with fatal neurodegenerative disorders resulting from accumulation and propagation of infectious prion protein (PrP^Sc^) in the brain^1,15^. At this time, there are no treatments available for prion diseases although several strategies are actively being investigated including vaccines and other immunotherapies^16,17^.

Relative to other infectious agents such as viruses and bacteria, prions pose unique challenges in terms of natural immunity, immunotherapy, and vaccine development. The human immune system is well-equipped to monitor for, and mount a response against, non-self antigens, be them endogenous (e.g. cancer neoantigens) or exogenous (e.g. viral proteins), while sparing self-antigens (tolerance), processes for which Human Leukocyte Antigen (HLA) is critical. However, despite conformational differences, the amino acid sequence of both PrP^C^ and PrP^Sc^ are identical; thus, activation of immune system responses targeting infectious agents is hampered by immune tolerance to self-proteins (PrP^C^ in this case), a challenge not only for natural immunity but also for development of prophylactic or treatment approaches for prion diseases^16,17^. Nonetheless, antibodies against PrP^C^ have been documented in humans without signs of prion pathology, indicating escape from self-tolerance and innocuous anti-PrP^C^ autoimmunity in some cases^18,19^. Moreover, several neuroprotective PrP^C^-binding antibody fragments have been identified from human antibody repertoires suggesting that anti-PrP^C^ antibodies exist in the population, contrary to expectations related to self-tolerance^18^. Overcoming self-tolerance via antigen selection and optimization may be a promising and plausible avenue for developing therapeutic strategies against prion diseases^16^. Since PrP^Sc^ requires PrP^C^ for propagation, reduction of PrP^C^ via antibodies is an appealing strategy^18^.

HLA molecules are cell-surface glycoproteins that work in concert to eliminate non-self proteins, including, e.g. proteins of pathogens (e.g. viral/bacterial) or neoantigens of cancer^20^. HLA molecules belong to two major classes, Class I (HLA-I) and Class II (HLA-II). Both HLA-I and HLA-II molecules are cell surface glycoproteins that present protein peptides to T cells. With respect to HLA Class I system, HLA-I molecules (encoded by the classical A, B, C genes) are expressed in all nucleated cells and produce molecules that bind with high affinity short peptides (mostly 9-mer) generated by the degradation of mostly endogenous proteins in the proteasome. The stable peptide-HLA-I complex (pHLA-I) moves to the cell surface where it is presented to circulating CD8+ T cells. CD8+ T cells that recognize the specific pHLA complex are activated and destroy cells that contain the non-self protein via various mechanisms, hence their direct cytotoxicity. With respect to HLA Class II system, HLA-II molecules (encoded by the classical DPB1, DQB1 and DRB1 genes) are expressed in specialized antigen presenting cells (e.g. macrophages, dendritic cells) and bind with high affinity longer peptides (mostly 15-mer) generated by the degradation of mostly exogenous proteins in the endo-lysosome compartment. The stable pHLA-II complex moves to the cell surface where it is presented to circulating CD4+ T cells which engage the B cells for production of antibodies against the offending protein but also enhance the activation of CD8+ T cells and also possess cytotoxic properties themselves. The cross-presentation pathway allows for processing of endogenous and exogenous antigens by both HLA-I and HLA-II systems. The HLA region is the most polymorphic in the human genome^21^; consequently, there is tremendous variability in HLA composition across individuals. The HLA composition of each individual determines the repertoire of antigens that can bind with sufficient affinity to promote an immune response^22–25^. Although the large HLA polymorphism almost guarantees survival at the population level, each individual carries only 12 HLA alleles, 2 per classical genes of HLA-I and HLA-II, which means that the success of the individual in dealing with/eliminating infectious and non-self antigens will be restricted, depending on the individual’s HLA genetic makeup.

Rejecting non-self proteins presupposes that those can be distinguished from self proteins. The mechanisms by which self proteins are recognized as such and are not attacked by the immune system (“immune tolerance”) are fairly complex and incompletely understood^26^. It is widely believed that escape from immune tolerance is a major contributing factor to autoimmune disorders^27,28^, with a recent estimated prevalence of 4.6% in the United States^29^. The HLA immunogenetic makeup is the main genetic factor underlying escape from immune tolerance^30^. Although it is commonly assumed that escape from immune tolerance, i.e. attacking self-proteins, is detrimental to health, this need not be universally true, since the health outcome would depend on the self protein being attacked. Naturally, we assume that all naturally occurring proteins in the body are “good”, and that is correct assuming the protein in question stays in its original configuration. The case in point is PRP^C^: in its natural form it is useful and innocuous but when (for ill-understood reasons) it misfolds, it transforms into the infectious and deadly PRP^Sc^. In this case, escape from immune tolerance against PRP^C^ could be beneficial, as it would reduce the number of PRP^C^ available for converting to PRP^Sc^. Interestingly, although PRP^C^ is widely expressed in many tissues, its absence in knockout mice lacking the PRNP gene has not been associated with serious health issues^9^. Escape of PRP^C^ from immune tolerance has been indicated by the reported existence of anti-PrP^C^ antibodies^18^, for which the HLA-II system would be involved. To our knowledge, there has been no systematic evaluation of HLA-related escape of PRP^C^ from immune tolerance. Here, we assessed *in silico* the predicted binding affinity of 334 HLA molecules (142 HLA-I and 192 HLA-II) with PrP^C^ peptides to search for and identify those capable of strong pHLA binding. Such molecules would underlie the hypothetical escape of PRP^C^ from immune tolerance, leading to reduction of PRP^C^ numbers directly (via CD8+ T cell activation, enhanced by CD4+ T cell activation) and/or indirectly (via CD4+ T cell activation of B cells for the production of anti-PRP^C^ antibodies).

## Materials and Methods

### Human prion protein (PRP^C^)

The amino acid (AA) sequence of the human major prion protein (PRP gene) was retrieved from the Uniprot database (https://www.uniprot.org/) on September 10, 2025 and is given in Table 1.

### HLA alleles

We investigated 142 HLA-I and 192 HLA-II common alleles^31^ shown in Tables S2 and S3, respectively.

### *In silico* determination of Predicted Binding Affinities PRP^C^

**Fig. 1.**
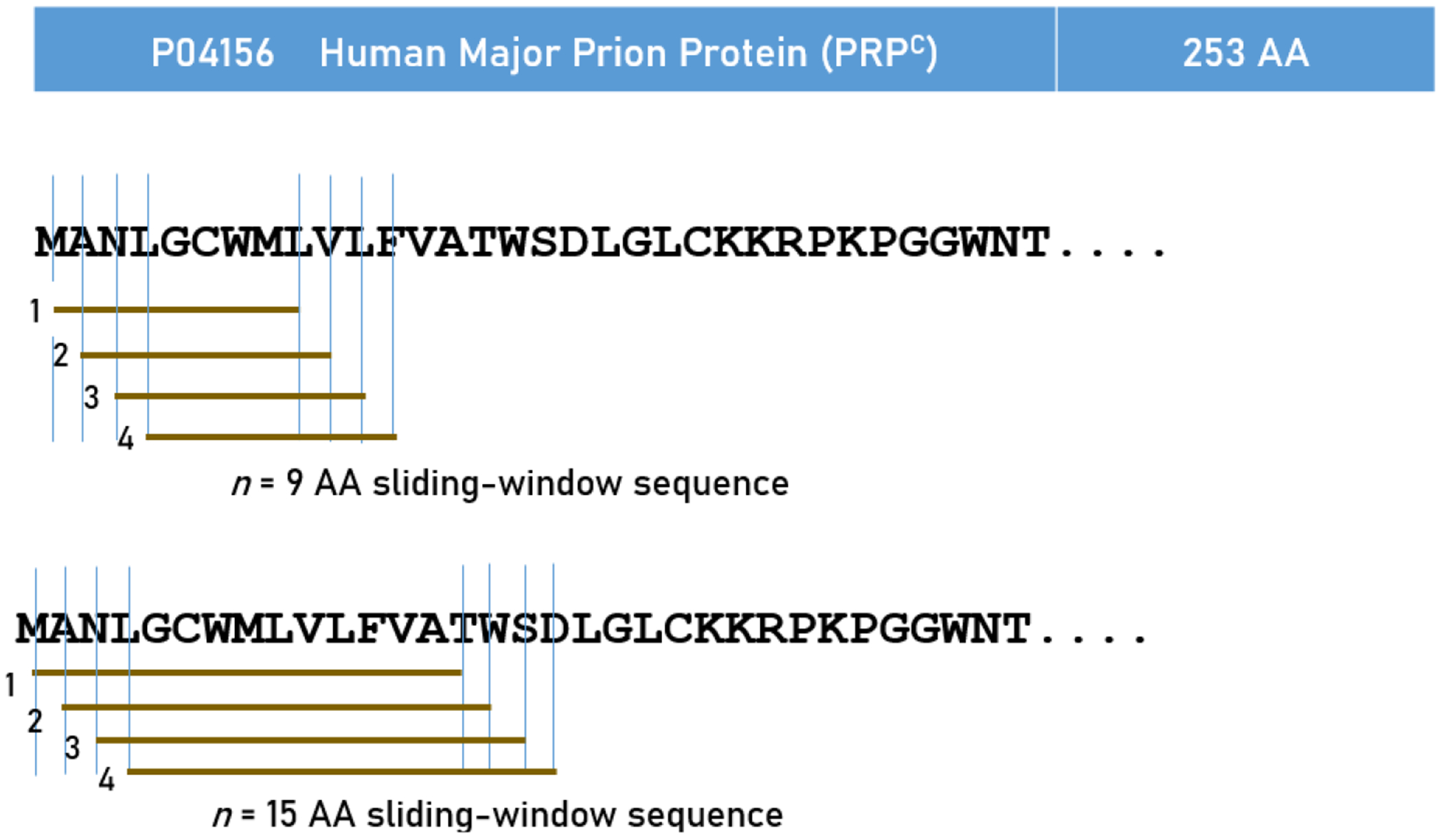
Schematic diagram to illustrate the sliding window approach for exhaustive testing of all consecutive linear 9-mer (A) and 15-mer peptides (B) of PRP^C^.

Predicted binding affinities were obtained for antigen peptides using the Immune Epitope Database (IEDB) NetMHCpan (ver. 4.1) tool^32,33^; accessed on September 12, 2025. More specifically, we used the sliding window approach^34–36^ to test exhaustively all possible linear 9-mer peptides for HLA-I predictions and 15-, 18- 22-mer peptides for HLA-II predictions. The method is illustrated in Fig. 1 for 9-mer and 15-mer peptides of PRP^C^. For each pair of peptide-HLA molecule tested, this tool gave, as an output, the IC_50_ of the predicted binding affinity; *the smaller the IC_50_, the stronger the binding affinity*. An IC_50_ value of < 50 nM (nanomolar) was regarded strong and 50 nM < IC_50_ < 500 nM values were regarded moderate^37^. Given a protein of N amino acid length and a peptide length of k AA, there are N-k+1 binding affinity predictions returned by the prediction tool. The numbers of 9-, 15, 18-, and 22-mer peptides tested are given in Table 2.

### Statistical analyses

The IBM-SPSS statistical package (version 30.0.0.0 172) was used for implementing statistical analyses. Standard statistical methods were used; all correlations are Pearson. All P-values reported are 2-sided, α = 0.05.

## Results

### Predicted binding affinities

We investigated 142 HLA-I alleles (41 HLA-A, 29 HLA-B, 72 HLA-C) (Table S1) and 192 HLA-II alleles (41 HLA-DPB1, 35 HLA-DQB1, 116 HLA-DRB1) (Table S2). The numbers of peptide-HLA allele complexes (pHLA) tested are shown in Table 2, together with the numbers and percentages of predicted strong and moderate binding affinities of 9-, 15-, 18-, and 22-mer peptides to HLA molecules. As expected, overall strong affinities were observed less frequently than moderate ones (0.369% vs. 4.305%). Details of pHLA complexes binding with high affinity are given in Tables S3–S6.

### Peptides

**Fig. 2.**
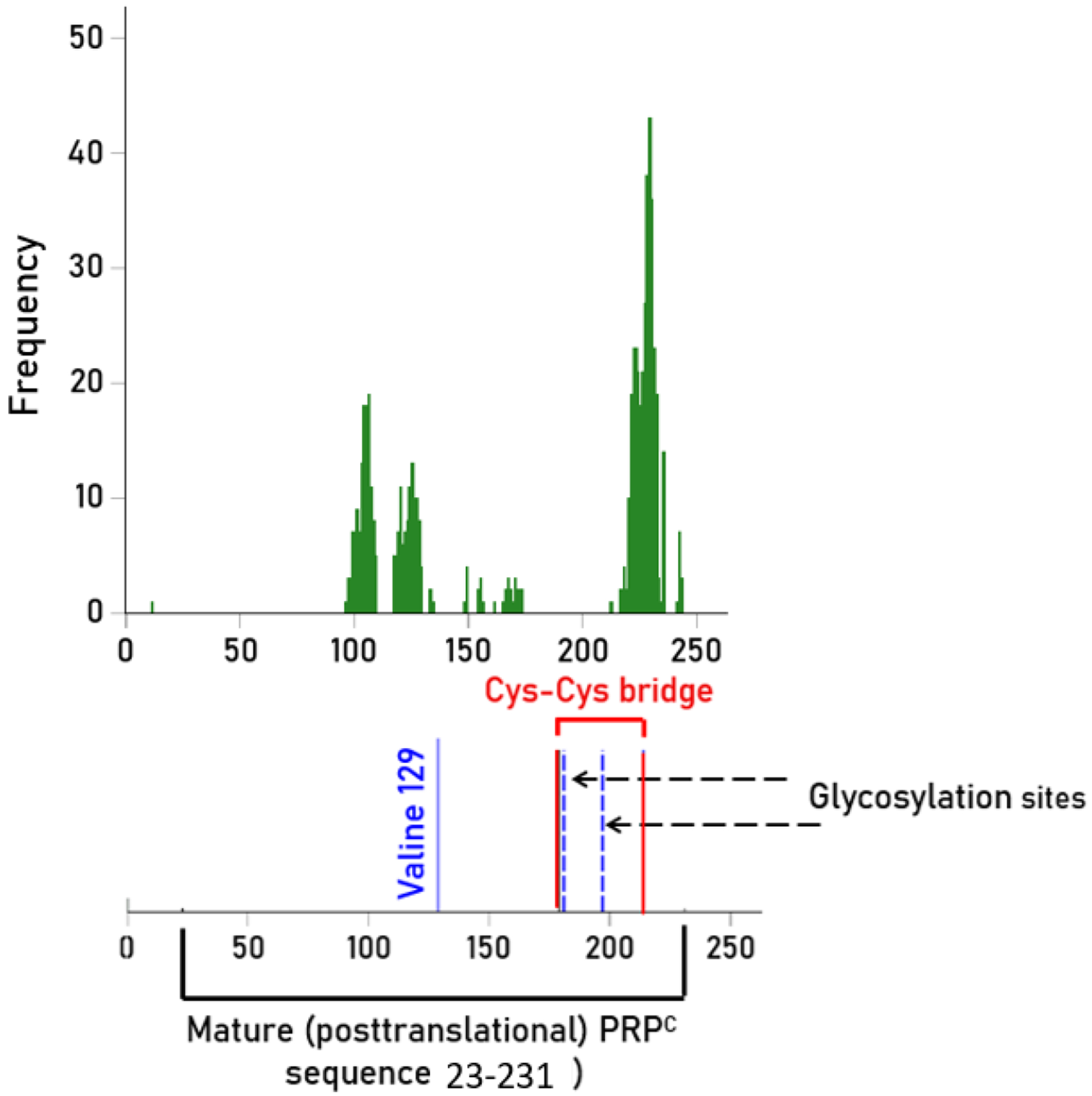
Bar graph shows the locations of the start of peptide sequences with predicted high binding affinity to all HLA molecules along the PRP^C^. N = 629 peptide locations from Tables S3–S6.

Of the total 952 PRP^C^ peptides tested (Table 2), 116 (12.18%) distinct peptides showed strong predicted binding affinity (IC_50_ < 50 nM) to HLA molecules and are shown in Table 3, amounting to a total of 629 peptides (given that they bound to more than one HLA molecule). The location of these peptides identified to have strong predicted bindings to any of the HLA tested (Tables S3–S6) is shown in Fig. 2. We found that 271/629 (43.1%) peptides were located within the mature (posttranslational) PRP^C^ (residues 23-231), arranged in 3 clusters. Overall, as can be seen more precisely in Tables S3–S6, several peptides contained the protective residue 129 (valine), glycosylation residues 181 and 197, and stabilizing Cys-Cys bridge residues 179 and 214. Antibodies against those peptides could potentially destabilize PRP^C^ making it prone to misfolding, an issue we discuss further in the Discussion section below.

### Alleles

**Fig. 3.**
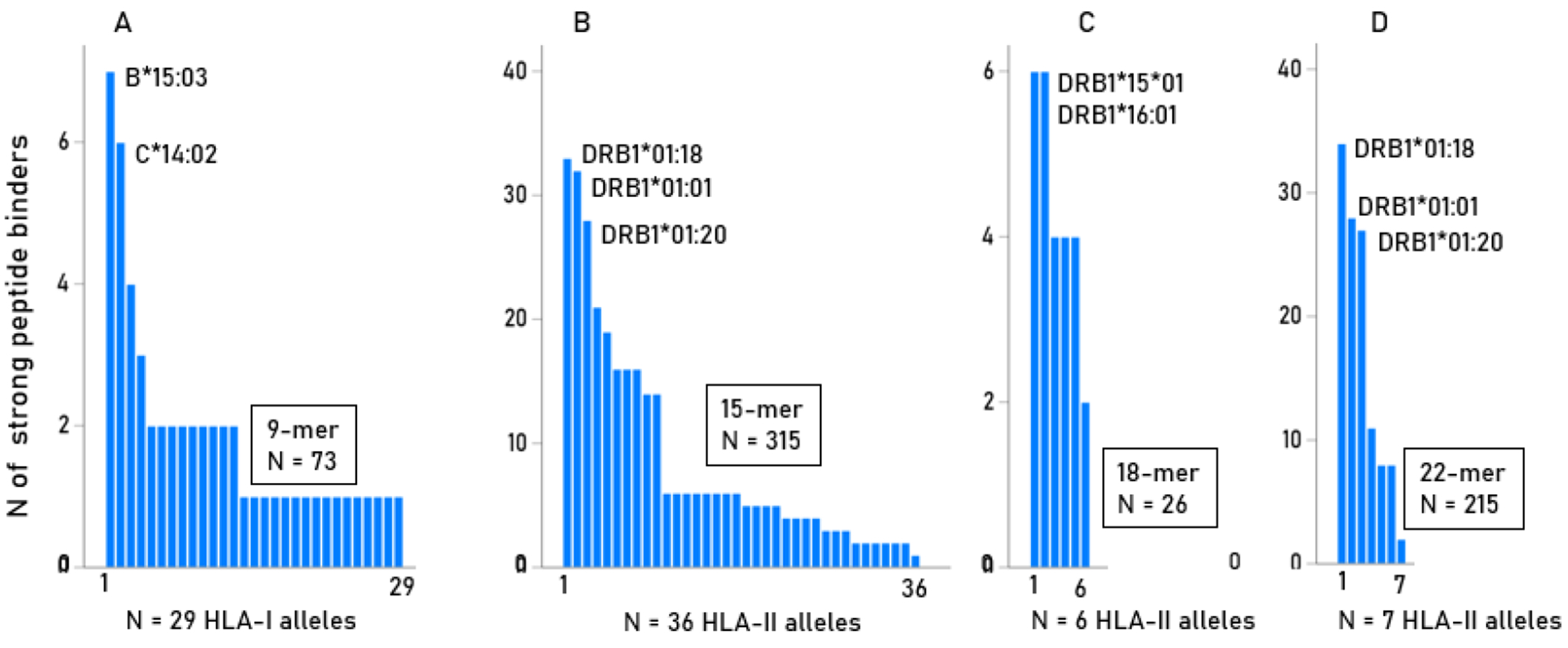
The counts of strongly binding peptides are plotted against alleles in a decreasing order. A, 9-mer peptides, HLA-I alleles; B, 15-mer peptides, HLA-II alleles; C, 18-mer peptides, HLA-II alleles; D, 22-mer peptides, HLA-II alleles. The bars in the X-axis indicate alleles in Table 2 plotted in the same order.

With respect to HLA-I, 29 (20.4%) alleles of the 142 tested, showed strong binding to at least one of the 245 peptides (9-mer) tested (range 1-7; Table 2; Fig.3A) and they were spread across all 3 HLA-I genes (A, B, C). In contrast, all strongly binding HLA-II molecules were confined to the DRB1 gene. More specifically, of the 192 alleles tested, (a) 36 (18.7%) showed strong binding to at least one of the 239 15-mer peptides tested (range 1-33; Table 2, Fig. 3B), (b) 6 (3.1%) showed strong binding to at least one of the 236 18-mer peptides tested (range 2-6; Table 2, Fig. 3C), and (c) 7 (3.6%) showed strong binding to at least one of the 239 22-mer peptides tested (range 2-34; Table 2, Fig. 3D). These results show that 15-mer peptides were the most effective HLA-II binders regarding both the total number of strong binders (N = 315) and spread among 36 alleles, whereas 18-mer peptides were the least effective (N = 26 strong binders among 6 alleles). Interestingly, 22-mer peptides were also effective binders (N = 119) but were spread only across 7 alleles. Since PRP^C^ is a natural, host protein, the presence of strong binding in all peptide lengths tested (9, 15, 18, 22-mer), although at different proportions, indicate evasion of tolerance during thymic selection.

### Overlap with other human proteins

We tested for possible overlap of the 116 peptides above to other human proteins by comparing them against the human proteome dataset version 24.1, provided by The Human Protein Atlas [The Human Protein Atlas. Accessed on October 22, 2025. https://www.proteinatlas.org/about/download#protein_atlas_data], comprising a total of 83607 human proteins.] They occurred only in 2 variants of the canonical PRP^C^ human protein (UNIPROT accession number P04156; 253 AA), namely truncated P04156 human prion proteins with accession numbers A2A2V1 (249 AA) and X6RKS3 (217 AA) with assumed similar function. Hence, involvement of HLA-II (15-mer, CD4+, antibody production) is restricted to PRP^C^ proteins only.

## Discussion

Misfolding of PRP^C^ to infectious PRP^Sc^ results in fatal neurodegeneration due to the accumulation of PrP^SC^ and lack of available treatment. It has been recognized that reduction of PrP^C^ via antibodies is an appealing strategy as it would reduce the number of PrP^C^ molecules available to convert to PrP^Sc 17,18^. Although self-tolerance to PrP^C^ may limit antibody production, recent reports suggest that, for some individuals, natural immune system responses overcome self-tolerance as evidenced by detection of PrP^C^ autoantibodies in the general population in the absence of any disease-specific association^18,19^. HLA is instrumental in the production of antibodies (via the CD4+ T cells of the HLA-II system) and for attacking and eliminating non-self proteins by direct destruction (via the CD8+ T cells of the HLA-I system, aided by the CD4+ helper T cells). With respect to HLA and autoimmunity, escape from immune tolerance can involve either or both of the HLA (Class I and II) systems. Here we evaluated both systems. The overall percentage of predicted high affinity binding was below 1% for all tests (Table 2), in keeping with similar HLA-I estimates for the whole human proteome^37^. Strongly binding HLA molecules were observed in 41/142 (28.9%) HLA-I alleles tested and occurred in all 3 genes (A, B, C; Table 4). In contrast, there were 36/192 (18.7%) strong HLA-II binders, all from the DRB1 gene. Since strong HLA binding would result in destruction of PRP^C^, our findings suggest that carriers of certain HLA alleles that bind strongly to PrP^C^ may have enhanced protection against prion disease, reflecting “good” autoimmunity in the sense that strong HLA-peptide binding affinity may reduce the number of potential PRP^C^ misfoldings by reducing the number of available PRP^C^ molecules available. In contrast, individuals lacking HLA molecules capable of strong PrP^C^ peptide binding may be at greater risk of developing prion disease due to reduced ability to mount an immune response aimed at PrP^C^ elimination. It is noteworthy that PRP^C^ is cleaved in the proteasome^38^ and, therefore, its 9-mer peptides can be presented to HLA-I alleles. Proteasome activity is inhibited by PRP^Sc 38^, contributing to PRP^Sc^ accumulation in the cell. In addition, PRP^C^ and PRP^Sc^ are degraded in the lysosome^39^, hence providing longer peptides (15-mer) for presentation to HLA-II molecules. Therefore, both HLA-I and HLA-II classes would contribute to limiting the number of PRP^C^ molecules available for misfolding. In addition, HLA-II molecules could be directly involved in lowering the PRP^Sc^ numbers via (a) reduction of PRP^C^ supply directly (via CD8+ T cell activation, enhanced by CD4+ T cell activation) and/or indirectly (via CD4+ T cell activation of B cells for the production of anti-PRP^C^ antibodies), and (b) destruction of PRP^Sc^ via CD4+ activation.

The current findings extend beyond natural immunity and point to specific peptides that may be useful for vaccine development (Table 3). Identification of immunogenic PrP^C^ peptides is an active line of research in pursuit of vaccines for prion diseases^16^. It is worth pointing out that immunogenicity of PrP^C^ peptides depends on HLA binding. Given the extreme heterogeneity of HLA^21^ and the effect of single amino acid differences on binding affinity^23^, the immunogenicity of a given PrP^C^ peptide is specific to a given HLA molecule. Each individual possesses a limited repertoire of HLA alleles that code for cell-surface HLA molecules. Here, we identified specific PrP^C^ peptide sequences that are predicted to bind strongly to a given a specific HLA molecule and could be considered for vaccine development. That being said, the translational potential of these findings rests on experimental validation including in vitro validation of binding assays, antigen presentation, and engagement of CD8+/CD4+ T cells and B-cells as well as in vivo validation in animal models and human epidemiological data. Such validation studies are particularly important in light of potential model error of *in silico* predictions, the possibility of destabilizing effects of antibody binding to glycosylation sites^40,41^ and cysteine bridge residues^42,43^, and evidence of potential neurotoxicity resulting from the interaction of antibodies with specific domains of PrP^C44^.

Finally, the findings here may hold relevance for other neurodegenerative conditions. Prion-like misfolding and aggregation of proteins including amyloid-β, tau, α-synuclein, and superoxide dismutase 1 have been implicated in the pathophysiology associated with Alzheimer’s disease, tauopathies, Parkinson’s disease, and amyotrophic lateral sclerosis, respectively^45–47^. HLA has also been implicated in risk/protection associated with various neurodegenerative diseases^48,49^. Based on the current findings, it is possible that documented HLA-related protection is partially related to HLA-mediated binding and elimination of misfolded proteins before they accumulate and lead to disease, a hypothesis that remains to be investigated.

## Supplementary Material

1

## Figures and Tables

**Table 1.** Amino acid sequence of PRP^J^ protein.

UniProt ID: P04156	Human major prion protein PRP^C^ (PRNP gene)	253 AA
MANLGCWMLVLFVATWSDLGLCKKRPKPGGWNTGGSRYPGQGSPGGNRYPPQGGGGWGQPHGGGWGQPHGGGWGQPHGGGWGQPHGGGWGQGGGTDSQWNKPSKPKTNMKHMAGAAAAGAVVGGLGGYMLGSAMSRPIIHFGSDYEDRYYRENMHRYPNQVYYRPMDEYSNQNNFVHDCVNITIKQHTVTTTTKGENFTETDVKMMERVVEQMCITQYERESQAYYQRGSSMVLFSSPPVILLISFLIFLIVG		

**Table 2.** Counts (and percentages) of pHLA tested and their predicted binding affinities.

	N Tested			N (%) Predicted binding affinity	
HLA	Peptides	Alleles	Total	Strong IC_01_ < 50 nM	Moderate 50 nM ≤ IC_01_ < 500 nM
HLA-I (9-mer)	245	142	34,790	73 (0.210%)	295 (0.848%)
HLA-II (15-mer)	239	192	45,888	315 (0.686)	2966 (6.464)
HLA-II (18-mer)	236	192	45,312	26 (0.057)	895 (1.975)
HLA-II (22-mer)	232	192	44,544	215 (0.483)	3186 (7.152)
Total	952		170,534	629 (0.369)	7342 (4.305)

**Table 3.** The 116 unique PRP^C^ peptides binding with high affinity (IC_50_ < 50 nM) to HLA-I and HLA-II molecules.

#	9-mer	#	15-mer	#	18-mer	#	22-mer
1	MVLFSSPPV	1	AAGAVVGGLGGYMLG	1	GSSMVLFSSPPVILLISF	1	AAGAVVGGLGGYMLGSAMSRPI
2	LLISFLIFL	2	AGAVVGGLGGYMLGS	2	KPKTNMKHMAGAAAAGAV	2	AGAVVGGLGGYMLGSAMSRPII
3	LISFLIFLI	3	AVVGGLGGYMLGSAM	3	KTNMKHMAGAAAAGAVVG	3	AVVGGLGGYMLGSAMSRPIIHF
4	VLFSSPPVI	4	AYYQRGSSMVLFSSP	4	PKTNMKHMAGAAAAGAVV	4	AYYQRGSSMVLFSSPPVILLIS
5	YYQRGSSMV	5	ESQAYYQRGSSMVLF	5	QRGSSMVLFSSPPVILLI	5	DEYSNQNNFVHDCVNITIKQHT
6	AYYQRGSSM	6	GAVVGGLGGYMLGSA	6	RGSSMVLFSSPPVILLIS	6	ERESQAYYQRGSSMVLFSSPPV
7	AVVGGLGGY	7	GGLGGYMLGSAMSRP	7	SKPKTNMKHMAGAAAAGA	7	ESQAYYQRGSSMVLFSSPPVIL
8	YYRENMHRY	8	GGYMLGSAMSRPIIH	8	SSMVLFSSPPVILLISFL	8	EYSNQNNFVHDCVNITIKQHTV
9	MSRPIIHFG	9	GLGGYMLGSAMSRPI	9	YQRGSSMVLFSSPPVILL	9	GAVVGGLGGYMLGSAMSRPIIH
10	KTNMKHMAG	10	GSSMVLFSSPPVILL	10	YYQRGSSMVLFSSPPVIL	10	GGLGGYMLGSAMSRPIIHFGSD
11	HSQWNKPSK	11	GYMLGSAMSRPIIHF			11	GLGGYMLGSAMSRPIIHFGSDY
12	RYYRENMHR	12	KPKTNMKHMAGAAAA			12	GSSMVLFSSPPVILLISFLIFL
13	RYPNQVYYR	13	KTNMKHMAGAAAAGA			13	KPKTNMKHMAGAAAAGAVVGGL
14	QMCITQYER	14	LGGYMLGSAMSRPII			14	KPSKPKTNMKHMAGAAAAGAVV
15	KPSKPKTNM	15	MKHMAGAAAAGAVVG			15	KTNMKHMAGAAAAGAVVGGLGG
16	YQRGSSMVL	16	MLGSAMSRPIIHFGS			16	LGGYMLGSAMSRPIIHFGSDYE
17	AMSRPIIHF	17	MVLFSSPPVILLISF			17	MDEYSNQNNFVHDCVNITIKQH
18	MHRYPNQVY	18	NMKHMAGAAAAGAVV			18	NKPSKPKTNMKHMAGAAAAGAV
19	YERESQAYY	19	NNFVHDCVNITIKQH			19	NMKHMAGAAAAGAVVGGLGGYM
20	HRYPNQVYY	20	NQNNFVHDCVNITIK			20	PKTNMKHMAGAAAAGAVVGGLG
21	MKHMAGAAA	21	PKTNMKHMAGAAAAG			21	PMDEYSNQNNFVHDCVNITIKQ
22	ILLISFLIF	22	QAYYQRGSSMVLFSS			22	PSKPKTNMKHMAGAAAAGAVVG
23	FSSPPVILL	23	QNNFVHDCVNITIKQ			23	QAYYQRGSSMVLFSSPPVILLI
24	DEYSNQNNF	24	QRGSSMVLFSSPPVI			24	QRGSSMVLFSSPPVILLISFLI
25	VYYRPMDEY	25	RESQAYYQRGSSMVL			25	QWNKPSKPKTNMKHMAGAAAAG
26	LFVATWSDL	26	RGSSMVLFSSPPVIL			26	QYERESQAYYQRGSSMVLFSSP
27	LFSSPPVIL	27	SKPKTNMKHMAGAAA			27	RESQAYYQRGSSMVLFSSPPVI
		28	SMVLFSSPPVILLIS			28	RGSSMVLFSSPPVILLISFLIF
		29	SNQNNFVHDCVNITI			29	SKPKTNMKHMAGAAAAGAVVGG
		30	SQAYYQRGSSMVLFS			30	SNQNNFVHDCVNITIKQHTVTT
		31	SSMVLFSSPPVILLI			31	SQAYYQRGSSMVLFSSPPVILL
		32	TNMKHMAGAAAAGAV			32	SQWNKPSKPKTNMKHMAGAAAA
		33	VGGLGGYMLGSAMSR			33	SSMVLFSSPPVILLISFLIFLI
		34	VLFSSPPVILLISFL			34	TNMKHMAGAAAAGAVVGGLGGY
		35	YMLGSAMSRPIIHFG			35	TQYERESQAYYQRGSSMVLFSS
		36	YQRGSSMVLFSSPPV			36	VGGLGGYMLGSAMSRPIIHFGS
		37	YYQRGSSMVLFSSPP			37	VVGGLGGYMLGSAMSRPIIHFG
						38	WNKPSKPKTNMKHMAGAAAAGA
						39	YERESQAYYQRGSSMVLFSSPP
						40	YQRGSSMVLFSSPPVILLISFL
						41	YSNQNNFVHDCVNITIKQHTVT
						42	YYQRGSSMVLFSSPPVILLISF

**Table 4.** Counts (N) of peptides with strong binding affinity (IC_50_ < 50 nM) to the listed HLA-I and HLA-II alleles (ranked from high to low counts).

9-mer	15-mer	18-mer	22-mer				
Allele	N strong	Allele	N strong	Allele	N strong	Allele	N strong
B*15:03	7	DRB1*01:18	33	DRB1*15:01	6	DRB1*01:18	34
C*14:02	6	DRB1*01:01	32	DRB1*15:06	6	DRB1*01:01	28
A*02:35	4	DRB1*01:20	28	DRB1*01:01	4	DRB1*01:20	27
A*30:01	3	DRB1*01:29	21	DRB1*01:18	4	DRB1*01:24	11
A*31:01	3	DRB1*01:24	19	DRB1*01:20	4	DRB1*01:02	8
C*07:02	3	DRB1*01:11	16	DRB1*15:02	2	DRB1*01:29	8
A*02:01	2	DRB1*07:01	16	Total	26	DRB1*16:02	2
A*02:02	2	DRB1*09:01	16			Total	118
A*02:06	2	DRB1*01:02	14				
A*02:30	2	DRB1*10:01	14				
A*02:63	2	DRB1*11:14	6				
A*02:77	2	DRB1*13:02	6				
A*24:03	2	DRB1*13:23	6				
B*15:01	2	DRB1*13:97	6				
B*18:01	2	DRB1*15:01	6				
C*03:02	2	DRB1*15:06	6				
C*03:03	2	DRB1*15:07	6				
C*03:04	2	DRB1*16:02	6				
A*02:05	1	DRB1*15:02	5				
A*26:01	1	DRB1*15:03	5				
A*26:08	1	DRB1*15:15	5				
A*29:01	1	DRB1*15:37	5				
A*29:02	1	DRB1*04:01	4				
A*30:02	1	DRB1*04:72	4				
A*68:02	1	DRB1*13:96	4				
B*07:02	1	DRB1*14:32	4				
B*07:05	1	DRB1*04:10	3				
B*15:17	1	DRB1*16:05	3				
B*15:18	1	DRB1*16:09	3				
B*39:02	1	DRB1*04:04	2				
C*07:01	1	DRB1*11:02	2				
C*12:03	1	DRB1*11:65	2				
C*15:02	1	DRB1*13:01	2				
C*15:04	1	DRB1*14:01	2				
C*15:05	1	DRB1*14:54	2				
C*15:06	1	DRB1*16:01	1				
C*15:09	1	Total	315				
C*16:01	1						
C*16:02	1						
C*16:04	1						
C*17:01	1						
Total	73						

## Data Availability

All data used were retrieved from freely accessible websites and, as such, are publicly and freely available [ref. [33]: http://tools.iedb.org/mhci/].
