# Supplementary material for: Predicted high affinity binding of prion PRPC protein to Human Leukocyte Antigen (HLA)
Source: J Immunol Sci. Author manuscript; Available in PMC 2026 Jun 8. (PMC13245592)
Supplement: 1 [file NIHMS2163297-supplement-1.pdf]

Supplementary Tables

Table S1. List of the 142 HLA-I alleles used.

Index	Allele
1	HLA-A*01:01
2	HLA-A*02:01
3	HLA-A*02:02
4	HLA-A*02:05
5	HLA-A*02:06
6	HLA-A*02:17
7	HLA-A*02:30
8	HLA-A*02:35
9	HLA-A*02:63
10	HLA-A*02:77
11	HLA-A*03:01
12	HLA-A*03:02
13	HLA-A*03:81
14	HLA-A*11:01
15	HLA-A*11:02
16	HLA-A*23:01
17	HLA-A*24:02
18	HLA-A*24:03
19	HLA-A*25:01
20	HLA-A*26:01
21	HLA-A*26:08
22	HLA-A*26:12
23	HLA-A*29:01
24	HLA-A*29:02
25	HLA-A*30:01
26	HLA-A*30:02
27	HLA-A*30:04
28	HLA-A*31:01
29	HLA-A*32:01
30	HLA-A*33:01
31	HLA-A*33:03
32	HLA-A*34:01
33	HLA-A*34:02
34	HLA-A*36:01
35	HLA-A*66:01
36	HLA-A*68:01
37	HLA-A*68:02
38	HLA-A*68:37
39	HLA-A*74:01
40	HLA-A*74:03
41	HLA-A*80:01
42	HLA-B*07:02
43	HLA-B*07:04
44	HLA-B*07:05
45	HLA-B*08:01
46	HLA-B*13:02
47	HLA-B*14:01
48	HLA-B*14:02
49	HLA-B*14:03
50	HLA-B*15:01
51	HLA-B*15:03
52	HLA-B*15:07
53	HLA-B*15:09
54	HLA-B*15:10
55	HLA-B*15:16
56	HLA-B*15:17
57	HLA-B*15:18
58	HLA-B*15:24
59	HLA-B*15:35
60	HLA-B*18:01
61	HLA-B*18:09
62	HLA-B*27:02
63	HLA-B*27:04
64	HLA-B*27:05
65	HLA-B*27:07
66	HLA-B*27:08
67	HLA-B*27:10
68	HLA-B*35:01
69	HLA-B*35:02
70	HLA-B*35:03
71	HLA-B*35:08
72	HLA-B*35:17
73	HLA-B*37:01
74	HLA-B*38:01
75	HLA-B*39:01
76	HLA-B*39:02
77	HLA-B*39:05
78	HLA-B*39:06
79	HLA-B*39:24
80	HLA-B*40:01
81	HLA-B*40:02

82	HLA-B*41:01
83	HLA-B*41:02
84	HLA-B*42:02
85	HLA-B*44:02
86	HLA-B*44:03
87	HLA-B*44:04
88	HLA-B*44:05
89	HLA-B*44:07
90	HLA-B*44:27
91	HLA-B*45:01
92	HLA-B*47:01
93	HLA-B*48:01
94	HLA-B*48:07
95	HLA-B*49:01
96	HLA-B*50:01
97	HLA-B*50:02
98	HLA-B*51:01
99	HLA-B*51:02
100	HLA-B*51:07
101	HLA-B*51:09
102	HLA-B*52:01
103	HLA-B*53:01
104	HLA-B*54:01
105	HLA-B*55:01
106	HLA-B*56:01
107	HLA-B*57:01
108	HLA-B*57:02
109	HLA-B*57:03
110	HLA-B*58:01
111	HLA-B*58:02
112	HLA-B*59:01
113	HLA-B*81:01
114	HLA-C*01:02
115	HLA-C*02:02
116	HLA-C*02:10
117	HLA-C*03:02
118	HLA-C*03:03
119	HLA-C*03:04
120	HLA-C*04:01
121	HLA-C*05:01
122	HLA-C*06:02
123	HLA-C*07:01
124	HLA-C*07:02
125	HLA-C*07:04
126	HLA-C*07:19
127	HLA-C*08:01
128	HLA-C*08:02
129	HLA-C*08:03
130	HLA-C*12:02
131	HLA-C*12:03
132	HLA-C*14:02
133	HLA-C*15:02
134	HLA-C*15:04
135	HLA-C*15:05
136	HLA-C*15:06
137	HLA-C*15:09
138	HLA-C*16:01
139	HLA-C*16:02
140	HLA-C*16:04
141	HLA-C*17:01
142	HLA-C*18:01

Table S2. List of the 192 HLA-II alleles used.

Index	Allele
1	DPB1*01:01
2	DPB1*02:01
3	DPB1*02:02
4	DPB1*03:01
5	DPB1*04:01
6	DPB1*04:02
7	DPB1*05:01
8	DPB1*06:01
9	DPB1*09:01
10	DPB1*10:01
11	DPB1*104:01
12	DPB1*105:01
13	DPB1*11:01
14	DPB1*124:01
15	DPB1*126:01
16	DPB1*13:01
17	DPB1*14:01
18	DPB1*15:01
19	DPB1*16:01
20	DPB1*17:01
21	DPB1*19:01
22	DPB1*20:01

23	DPB1*23:01	66	DQB1*06:07
24	DPB1*26:01	67	DQB1*06:08
25	DPB1*28:01	68	DQB1*06:09
26	DPB1*30:01	69	DQB1*06:11
27	DPB1*33:01	70	DQB1*06:14
28	DPB1*34:01	71	DQB1*06:15
29	DPB1*35:01	72	DQB1*06:18
30	DPB1*39:01	73	DQB1*06:19
31	DPB1*40:01	74	DQB1*06:22
32	DPB1*41:01	75	DQB1*06:27
33	DPB1*46:01	76	DQB1*06:32
34	DPB1*47:01	77	DRB1*01:01
35	DPB1*49:01	78	DRB1*01:02
36	DPB1*55:01	79	DRB1*01:03
37	DPB1*71:01	80	DRB1*01:11
38	DPB1*72:01	81	DRB1*01:18
39	DPB1*81:01	82	DRB1*01:20
40	DPB1*85:01	83	DRB1*01:24
41	DPB1*91:01	84	DRB1*01:29
42	DQB1*02:02	85	DRB1*03:01
43	DQB1*03:01	86	DRB1*03:02
44	DQB1*03:02	87	DRB1*03:04
45	DQB1*03:03	88	DRB1*03:05
46	DQB1*03:04	89	DRB1*03:11
47	DQB1*03:05	90	DRB1*03:13
48	DQB1*03:10	91	DRB1*03:15
49	DQB1*03:14	92	DRB1*03:41
50	DQB1*03:17	93	DRB1*04:01
51	DQB1*03:19	94	DRB1*04:02
52	DQB1*03:23	95	DRB1*04:03
53	DQB1*03:25	96	DRB1*04:04
54	DQB1*04:01	97	DRB1*04:05
55	DQB1*04:02	98	DRB1*04:06
56	DQB1*04:03	99	DRB1*04:07
57	DQB1*05:01	100	DRB1*04:08
58	DQB1*05:02	101	DRB1*04:10
59	DQB1*05:03	102	DRB1*04:11
60	DQB1*05:06	103	DRB1*04:17
61	DQB1*05:11	104	DRB1*04:44
62	DQB1*06:01	105	DRB1*04:53
63	DQB1*06:02	106	DRB1*04:56
64	DQB1*06:03	107	DRB1*04:72
65	DQB1*06:04	108	DRB1*07:01

109	DRB1*08:01
110	DRB1*08:02
111	DRB1*08:03
112	DRB1*08:04
113	DRB1*08:24
114	DRB1*08:30
115	DRB1*08:36
116	DRB1*09:01
117	DRB1*09:02
118	DRB1*10:01
119	DRB1*11:01
120	DRB1*11:02
121	DRB1*11:03
122	DRB1*11:04
123	DRB1*11:06
124	DRB1*11:07
125	DRB1*11:08
126	DRB1*11:10
127	DRB1*11:11
128	DRB1*11:12
129	DRB1*11:13
130	DRB1*11:14
131	DRB1*11:19
132	DRB1*11:27
133	DRB1*11:28
134	DRB1*11:29
135	DRB1*11:37
136	DRB1*11:42
137	DRB1*11:46
138	DRB1*11:49
139	DRB1*11:54
140	DRB1*11:58
141	DRB1*11:62
142	DRB1*11:65
143	DRB1*11:74
144	DRB1*11:84
145	DRB1*12:01
146	DRB1*12:02
147	DRB1*12:03
148	DRB1*12:16
149	DRB1*13:01
150	DRB1*13:02
151	DRB1*13:03
152	DRB1*13:05
153	DRB1*13:07
154	DRB1*13:11
155	DRB1*13:12
156	DRB1*13:14
157	DRB1*13:21
158	DRB1*13:23
159	DRB1*13:26
160	DRB1*13:33
161	DRB1*13:50
162	DRB1*13:61
163	DRB1*13:66
164	DRB1*13:96
165	DRB1*13:97
166	DRB1*14:01
167	DRB1*14:02
168	DRB1*14:03
169	DRB1*14:04
170	DRB1*14:05
171	DRB1*14:06
172	DRB1*14:07
173	DRB1*14:12
174	DRB1*14:23
175	DRB1*14:27
176	DRB1*14:32
177	DRB1*14:38
178	DRB1*14:44
179	DRB1*14:54
180	DRB1*14:68
181	DRB1*15:01
182	DRB1*15:02
183	DRB1*15:03
184	DRB1*15:06
185	DRB1*15:07
186	DRB1*15:15
187	DRB1*15:37
188	DRB1*16:01
189	DRB1*16:02
190	DRB1*16:04
191	DRB1*16:05
192	DRB1*16:09

Table S3. Peptides (9-mer) of PRP^C binding with high affinity (IC_{50} < 50 nM) to HLA-I molecules.

Index	Allele	Start	End	Peptide	min(IC_{50})
1	A*02:01	232	240	MVLFSSPPV	19.28
2	A*02:01	242	250	LLISFLIFL	21.74
3	A*02:02	242	250	LLISFLIFL	21.62
4	A*02:02	243	251	LISFLIFLI	22.31
5	A*02:05	232	240	MVLFSSPPV	36.84
6	A*02:06	232	240	MVLFSSPPV	6.41
7	A*02:06	242	250	LLISFLIFL	45.43
8	A*02:30	232	240	MVLFSSPPV	19.28
9	A*02:30	242	250	LLISFLIFL	21.74
10	A*02:35	232	240	MVLFSSPPV	11.92
11	A*02:35	233	241	VLFSSPPVI	28.52
12	A*02:35	242	250	LLISFLIFL	33.02
13	A*02:35	243	251	LISFLIFLI	42.66
14	A*02:63	242	250	LLISFLIFL	21.62
15	A*02:63	243	251	LISFLIFLI	22.31
16	A*02:77	232	240	MVLFSSPPV	19.28
17	A*02:77	242	250	LLISFLIFL	21.74
18	A*24:03	225	233	YYQRGSSMV	17.43
19	A*24:03	224	232	AYYQRGSSM	32.21
20	A*26:01	120	128	AVVGGLGGY	18.35
21	A*26:08	120	128	AVVGGLGGY	22.27
22	A*29:01	149	157	YYRENMHRY	30.34
23	A*29:02	149	157	YYRENMHRY	30.34
24	A*30:01	134	142	MSRPIIHFG	20.52
25	A*30:01	106	114	KTNMKHMAG	24.40
26	A*30:01	96	104	HSQWNKPSK	33.00
27	A*30:02	120	128	AVVGGLGGY	22.99
28	A*31:01	148	156	RYYRENMHR	9.50
29	A*31:01	156	164	RYPNQVYYR	11.00
30	A*31:01	212	220	QMCITQYER	28.98
31	A*68:02	232	240	MVLFSSPPV	10.70
32	B*07:02	101	109	KPSKPKTNM	24.54
33	B*07:05	101	109	KPSKPKTNM	14.38
34	B*15:01	226	234	YQRGSSMVL	15.83
35	B*15:01	133	141	AMSRPIIHF	35.33
36	B*15:03	226	234	YQRGSSMVL	3.40
37	B*15:03	154	162	MHRYPNQVY	7.32
38	B*15:03	133	141	AMSRPIIHF	17.45
39	B*15:03	218	226	YERESQAYY	18.85
40	B*15:03	155	163	HRYPNQVYY	32.51
41	B*15:03	109	117	MKHMAGAAA	38.59
42	B*15:03	241	249	ILLISFLIF	47.91
43	B*15:17	235	243	FSSPPVILL	16.04
44	B*15:18	154	162	MHRYPNQVY	31.57
45	B*18:01	167	175	DEYSNQNNF	26.50
46	B*18:01	218	226	YERESQAYY	34.56
47	B*39:02	226	234	YQRGSSMVL	17.58
48	C*03:02	235	243	FSSPPVILL	19.16
49	C*03:02	226	234	YQRGSSMVL	35.20
50	C*03:03	235	243	FSSPPVILL	7.82
51	C*03:03	226	234	YQRGSSMVL	37.08
52	C*03:04	235	243	FSSPPVILL	7.82
53	C*03:04	226	234	YQRGSSMVL	37.08
54	C*07:01	155	163	HRYPNQVYY	32.07
55	C*07:02	149	157	YYRENMHRY	37.07
56	C*07:02	155	163	HRYPNQVYY	38.83
57	C*07:02	224	232	AYYQRGSSM	46.16
58	C*12:03	235	243	FSSPPVILL	12.15
59	C*14:02	224	232	AYYQRGSSM	2.49
60	C*14:02	149	157	YYRENMHRY	6.21
61	C*14:02	225	233	YYQRGSSMV	11.39
62	C*14:02	161	169	VYYRPMDEY	23.88
63	C*14:02	11	19	LFVATWSDL	29.66
64	C*14:02	234	242	LFSSPPVIL	33.27
65	C*15:02	235	243	FSSPPVILL	17.92
66	C*15:04	235	243	FSSPPVILL	23.04
67	C*15:05	235	243	FSSPPVILL	24.99
68	C*15:06	235	243	FSSPPVILL	11.97
69	C*15:09	235	243	FSSPPVILL	23.04
70	C*16:01	235	243	FSSPPVILL	11.54
71	C*16:02	235	243	FSSPPVILL	13.36
72	C*16:04	235	243	FSSPPVILL	16.02
73	C*17:01	235	243	FSSPPVILL	11.12

Table S4. Peptides (15-mer) of PRP^C binding with high affinity (IC_{50} < 50 nM) to HLA-II molecules.

Index	Allele	Start	End	Peptide	IC_{50}
1	DRB1*01:01	106	120	KTNMKHMAGAAAAGA	6.93
2	DRB1*01:01	223	237	QAYYQRGSSMVLFSS	7.40
3	DRB1*01:01	105	119	PKTNMKHMAGAAAAG	7.65
4	DRB1*01:01	104	118	KPKTNMKHMAGAAAA	7.99
5	DRB1*01:01	222	236	SQAYYQRGSSMVLFS	8.36
6	DRB1*01:01	107	121	TNMKHMAGAAAAGAV	8.50
7	DRB1*01:01	108	122	NMKHMAGAAAAGAVV	10.72
8	DRB1*01:01	224	238	AYYQRGSSMVLFSSP	11.13
9	DRB1*01:01	221	235	ESQAYYQRGSSMVLF	11.35
10	DRB1*01:01	127	141	GYMLGSAMSRPIIHF	12.29
11	DRB1*01:01	126	140	GGYMLGSAMSRPIIH	13.74
12	DRB1*01:01	125	139	LGGYMLGSAMSRPII	14.53
13	DRB1*01:01	230	244	SSMVLFSSPPVILLI	15.75
14	DRB1*01:01	231	245	SMVLFSSPPVILLIS	16.06
15	DRB1*01:01	229	243	GSSMVLFSSPPVILL	16.19
16	DRB1*01:01	124	138	GLGGYMLGSAMSRPI	18.61
17	DRB1*01:01	128	142	YMLGSAMSRPIIHFG	19.17
18	DRB1*01:01	103	117	SKPKTNMKHMAGAAA	21.12
19	DRB1*01:01	119	133	GAVVGGLGGYMLGSA	22.16
20	DRB1*01:01	225	239	YYQRGSSMVLFSSPP	22.22
21	DRB1*01:01	228	242	RGSSMVLFSSPPVIL	22.52
22	DRB1*01:01	232	246	MVLFSSPPVILLISF	23.21
23	DRB1*01:01	109	123	MKHMAGAAAAGAVVG	24.72
24	DRB1*01:01	220	234	RESQAYYQRGSSMVL	25.35
25	DRB1*01:01	120	134	AVVGGLGGYMLGSAM	28.96
26	DRB1*01:01	123	137	GGLGGYMLGSAMSRP	28.97
27	DRB1*01:01	118	132	AGAVVGGLGGYMLGS	30.05
28	DRB1*01:01	122	136	VGGLGGYMLGSAMSR	36.63
29	DRB1*01:01	227	241	QRGSSMVLFSSPPVI	36.82
30	DRB1*01:01	233	247	VLFSSPPVILLISFL	41.55
31	DRB1*01:01	129	143	MLGSAMSRPIIHFGS	43.97
32	DRB1*01:01	117	131	AAGAVVGGLGGYMLG	46.16
33	DRB1*01:02	106	120	KTNMKHMAGAAAAGA	21.53
34	DRB1*01:02	127	141	GYMLGSAMSRPIIHF	23.12
35	DRB1*01:02	105	119	PKTNMKHMAGAAAAG	25.47
36	DRB1*01:02	104	118	KPKTNMKHMAGAAAA	27.77
37	DRB1*01:02	230	244	SSMVLFSSPPVILLI	28.07
38	DRB1*01:02	107	121	TNMKHMAGAAAAGAV	28.45
39	DRB1*01:02	229	243	GSSMVLFSSPPVILL	30.49
40	DRB1*01:02	231	245	SMVLFSSPPVILLIS	30.75
41	DRB1*01:02	128	142	YMLGSAMSRPIIHFG	31.72
42	DRB1*01:02	126	140	GGYMLGSAMSRPIIH	32.46
43	DRB1*01:02	108	122	NMKHMAGAAAAGAVV	37.20
44	DRB1*01:02	125	139	LGGYMLGSAMSRPII	40.18
45	DRB1*01:02	228	242	RGSSMVLFSSPPVIL	47.95
46	DRB1*01:02	119	133	GAVVGGLGGYMLGSA	48.50
47	DRB1*01:11	223	237	QAYYQRGSSMVLFSS	18.16
48	DRB1*01:11	222	236	SQAYYQRGSSMVLFS	20.57
49	DRB1*01:11	106	120	KTNMKHMAGAAAAGA	21.23
50	DRB1*01:11	224	238	AYYQRGSSMVLFSSP	27.11
51	DRB1*01:11	105	119	PKTNMKHMAGAAAAG	27.74
52	DRB1*01:11	221	235	ESQAYYQRGSSMVLF	27.98
53	DRB1*01:11	230	244	SSMVLFSSPPVILLI	28.76
54	DRB1*01:11	229	243	GSSMVLFSSPPVILL	29.84
55	DRB1*01:11	231	245	SMVLFSSPPVILLIS	30.40
56	DRB1*01:11	104	118	KPKTNMKHMAGAAAA	32.10
57	DRB1*01:11	107	121	TNMKHMAGAAAAGAV	35.42
58	DRB1*01:11	127	141	GYMLGSAMSRPIIHF	38.36
59	DRB1*01:11	232	246	MVLFSSPPVILLISF	44.43
60	DRB1*01:11	125	139	LGGYMLGSAMSRPII	44.56
61	DRB1*01:11	126	140	GGYMLGSAMSRPIIH	45.39
62	DRB1*01:11	228	242	RGSSMVLFSSPPVIL	46.12
63	DRB1*01:18	106	120	KTNMKHMAGAAAAGA	7.93
64	DRB1*01:18	223	237	QAYYQRGSSMVLFSS	8.43
65	DRB1*01:18	105	119	PKTNMKHMAGAAAAG	9.26
66	DRB1*01:18	222	236	SQAYYQRGSSMVLFS	9.36
67	DRB1*01:18	104	118	KPKTNMKHMAGAAAA	10.16
68	DRB1*01:18	107	121	TNMKHMAGAAAAGAV	10.37
69	DRB1*01:18	221	235	ESQAYYQRGSSMVLF	12.36
70	DRB1*01:18	127	141	GYMLGSAMSRPIIHF	12.43
71	DRB1*01:18	224	238	AYYQRGSSMVLFSSP	12.48
72	DRB1*01:18	229	243	GSSMVLFSSPPVILL	12.61
73	DRB1*01:18	230	244	SSMVLFSSPPVILLI	13.03
74	DRB1*01:18	126	140	GGYMLGSAMSRPIIH	13.32
75	DRB1*01:18	108	122	NMKHMAGAAAAGAVV	13.58
76	DRB1*01:18	125	139	LGGYMLGSAMSRPII	13.94
77	DRB1*01:18	231	245	SMVLFSSPPVILLIS	14.78
78	DRB1*01:18	228	242	RGSSMVLFSSPPVIL	16.69
79	DRB1*01:18	124	138	GLGGYMLGSAMSRPI	18.88
80	DRB1*01:18	128	142	YMLGSAMSRPIIHFG	19.71
81	DRB1*01:18	232	246	MVLFSSPPVILLISF	22.06
82	DRB1*01:18	119	133	GAVVGGLGGYMLGSA	23.48
83	DRB1*01:18	220	234	RESQAYYQRGSSMVL	25.66
84	DRB1*01:18	225	239	YYQRGSSMVLFSSPP	25.68
85	DRB1*01:18	227	241	QRGSSMVLFSSPPVI	26.17
86	DRB1*01:18	103	117	SKPKTNMKHMAGAAA	26.67
87	DRB1*01:18	120	134	AVVGGLGGYMLGSAM	28.47
88	DRB1*01:18	118	132	AGAVVGGLGGYMLGS	30.65
89	DRB1*01:18	123	137	GGLGGYMLGSAMSRP	32.26
90	DRB1*01:18	109	123	MKHMAGAAAAGAVVG	33.06
91	DRB1*01:18	233	247	VLFSSPPVILLISFL	39.27
92	DRB1*01:18	122	136	VGGLGGYMLGSAMSR	42.11
93	DRB1*01:18	117	131	AAGAVVGGLGGYMLG	44.28
94	DRB1*01:18	226	240	YQRGSSMVLFSSPPV	46.28
95	DRB1*01:18	129	143	MLGSAMSRPIIHFGS	48.50
96	DRB1*01:20	106	120	KTNMKHMAGAAAAGA	8.31
97	DRB1*01:20	105	119	PKTNMKHMAGAAAAG	9.36
98	DRB1*01:20	104	118	KPKTNMKHMAGAAAA	9.75
99	DRB1*01:20	107	121	TNMKHMAGAAAAGAV	10.18
100	DRB1*01:20	229	243	GSSMVLFSSPPVILL	11.23
101	DRB1*01:20	127	141	GYMLGSAMSRPIIHF	11.49
102	DRB1*01:20	230	244	SSMVLFSSPPVILLI	11.87
103	DRB1*01:20	108	122	NMKHMAGAAAAGAVV	12.12
104	DRB1*01:20	231	245	SMVLFSSPPVILLIS	13.29
105	DRB1*01:20	228	242	RGSSMVLFSSPPVIL	14.56

106	DRB1*01:20	126	140	GGYMLGSAMSRPIIH	14.73
107	DRB1*01:20	119	133	GAVVGGLGGYMLGSA	14.77
108	DRB1*01:20	125	139	LGGYMLGSAMSRPII	16.65
109	DRB1*01:20	128	142	YMLGSAMSRPIIHFG	16.75
110	DRB1*01:20	118	132	AGAVVGGLGGYMLGS	18.85
111	DRB1*01:20	120	134	AVVGGLGGYMLGSAM	19.21
112	DRB1*01:20	232	246	MVLFSSPPVILLISF	22.84
113	DRB1*01:20	227	241	QRGSSMVLFSSPPVI	23.38
114	DRB1*01:20	103	117	SKPKTNMKHMAGAAA	24.78
115	DRB1*01:20	124	138	GLGGYMLGSAMSRPI	25.19
116	DRB1*01:20	117	131	AAGAVVGGLGGYMLG	27.24
117	DRB1*01:20	109	123	MKHMAGAAAAGAVVG	30.35
118	DRB1*01:20	223	237	QAYYQRGSSMVLFSS	30.93
119	DRB1*01:20	222	236	SQAYYQRGSSMVLFS	31.98
120	DRB1*01:20	129	143	MLGSAMSRPIIHFGS	36.80
121	DRB1*01:20	121	135	VVGGLGGYMLGSAMS	37.26
122	DRB1*01:20	221	235	ESQAYYQRGSSMVLF	40.23
123	DRB1*01:20	123	137	GGLGGYMLGSAMSRP	43.03
124	DRB1*01:24	106	120	KTNMKHMAGAAAAGA	14.71
125	DRB1*01:24	223	237	QAYYQRGSSMVLFSS	16.19
126	DRB1*01:24	222	236	SQAYYQRGSSMVLFS	17.64
127	DRB1*01:24	105	119	PKTNMKHMAGAAAAG	17.83
128	DRB1*01:24	104	118	KPKTNMKHMAGAAAA	19.83
129	DRB1*01:24	107	121	TNMKHMAGAAAAGAV	21.38
130	DRB1*01:24	230	244	SSMVLFSSPPVILLI	22.94
131	DRB1*01:24	224	238	AYYQRGSSMVLFSSP	23.12
132	DRB1*01:24	221	235	ESQAYYQRGSSMVLF	23.48
133	DRB1*01:24	229	243	GSSMVLFSSPPVILL	24.22
134	DRB1*01:24	231	245	SMVLFSSPPVILLIS	24.73
135	DRB1*01:24	127	141	GYMLGSAMSRPIIHF	27.32
136	DRB1*01:24	108	122	NMKHMAGAAAAGAVV	32.50
137	DRB1*01:24	125	139	LGGYMLGSAMSRPII	36.15
138	DRB1*01:24	228	242	RGSSMVLFSSPPVIL	36.69
139	DRB1*01:24	126	140	GGYMLGSAMSRPIIH	37.21
140	DRB1*01:24	128	142	YMLGSAMSRPIIHFG	39.57
141	DRB1*01:24	232	246	MVLFSSPPVILLISF	40.00
142	DRB1*01:24	225	239	YYQRGSSMVLFSSPP	46.98
143	DRB1*01:29	106	120	KTNMKHMAGAAAAGA	13.48
144	DRB1*01:29	223	237	QAYYQRGSSMVLFSS	16.08
145	DRB1*01:29	105	119	PKTNMKHMAGAAAAG	16.80
146	DRB1*01:29	222	236	SQAYYQRGSSMVLFS	18.18
147	DRB1*01:29	104	118	KPKTNMKHMAGAAAA	19.55
148	DRB1*01:29	107	121	TNMKHMAGAAAAGAV	19.66
149	DRB1*01:29	127	141	GYMLGSAMSRPIIHF	23.35
150	DRB1*01:29	224	238	AYYQRGSSMVLFSSP	23.60
151	DRB1*01:29	221	235	ESQAYYQRGSSMVLF	24.51
152	DRB1*01:29	108	122	NMKHMAGAAAAGAVV	24.56
153	DRB1*01:29	230	244	SSMVLFSSPPVILLI	24.91
154	DRB1*01:29	231	245	SMVLFSSPPVILLIS	25.70
155	DRB1*01:29	229	243	GSSMVLFSSPPVILL	25.90
156	DRB1*01:29	126	140	GGYMLGSAMSRPIIH	27.73
157	DRB1*01:29	125	139	LGGYMLGSAMSRPII	30.43
158	DRB1*01:29	128	142	YMLGSAMSRPIIHFG	35.49
159	DRB1*01:29	232	246	MVLFSSPPVILLISF	37.79
160	DRB1*01:29	228	242	RGSSMVLFSSPPVIL	37.89
161	DRB1*01:29	124	138	GLGGYMLGSAMSRPI	43.18
162	DRB1*01:29	225	239	YYQRGSSMVLFSSPP	49.43
163	DRB1*01:29	109	123	MKHMAGAAAAGAVVG	49.99
164	DRB1*04:01	172	186	QNNFVHDCVNITIKQ	22.64
165	DRB1*04:01	171	185	NQNNFVHDCVNITIK	23.88
166	DRB1*04:01	173	187	NNFVHDCVNITIKQH	30.82
167	DRB1*04:01	170	184	SNQNNFVHDCVNITI	37.41
168	DRB1*04:04	229	243	GSSMVLFSSPPVILL	36.22
169	DRB1*04:04	228	242	RGSSMVLFSSPPVIL	42.31
170	DRB1*04:10	229	243	GSSMVLFSSPPVILL	39.61
171	DRB1*04:10	228	242	RGSSMVLFSSPPVIL	44.46
172	DRB1*04:10	230	244	SSMVLFSSPPVILLI	49.89
173	DRB1*04:72	172	186	QNNFVHDCVNITIKQ	25.13
174	DRB1*04:72	171	185	NQNNFVHDCVNITIK	26.85
175	DRB1*04:72	173	187	NNFVHDCVNITIKQH	34.26
176	DRB1*04:72	170	184	SNQNNFVHDCVNITI	42.02
177	DRB1*07:01	127	141	GYMLGSAMSRPIIHF	14.05
178	DRB1*07:01	230	244	SSMVLFSSPPVILLI	16.37
179	DRB1*07:01	126	140	GGYMLGSAMSRPIIH	16.61
180	DRB1*07:01	229	243	GSSMVLFSSPPVILL	16.64
181	DRB1*07:01	125	139	LGGYMLGSAMSRPII	17.75
182	DRB1*07:01	231	245	SMVLFSSPPVILLIS	17.78
183	DRB1*07:01	222	236	SQAYYQRGSSMVLFS	20.35
184	DRB1*07:01	228	242	RGSSMVLFSSPPVIL	20.68
185	DRB1*07:01	128	142	YMLGSAMSRPIIHFG	20.86
186	DRB1*07:01	223	237	QAYYQRGSSMVLFSS	21.18
187	DRB1*07:01	221	235	ESQAYYQRGSSMVLF	21.81
188	DRB1*07:01	232	246	MVLFSSPPVILLISF	26.93
189	DRB1*07:01	124	138	GLGGYMLGSAMSRPI	28.93
190	DRB1*07:01	220	234	RESQAYYQRGSSMVL	29.31
191	DRB1*07:01	224	238	AYYQRGSSMVLFSSP	33.65
192	DRB1*07:01	227	241	QRGSSMVLFSSPPVI	48.94
193	DRB1*09:01	127	141	GYMLGSAMSRPIIHF	14.17
194	DRB1*09:01	126	140	GGYMLGSAMSRPIIH	15.03
195	DRB1*09:01	125	139	LGGYMLGSAMSRPII	15.74
196	DRB1*09:01	223	237	QAYYQRGSSMVLFSS	19.76
197	DRB1*09:01	222	236	SQAYYQRGSSMVLFS	20.12
198	DRB1*09:01	128	142	YMLGSAMSRPIIHFG	20.37
199	DRB1*09:01	221	235	ESQAYYQRGSSMVLF	22.50
200	DRB1*09:01	124	138	GLGGYMLGSAMSRPI	23.44
201	DRB1*09:01	229	243	GSSMVLFSSPPVILL	27.57
202	DRB1*09:01	230	244	SSMVLFSSPPVILLI	27.99
203	DRB1*09:01	220	234	RESQAYYQRGSSMVL	29.39
204	DRB1*09:01	224	238	AYYQRGSSMVLFSSP	30.37
205	DRB1*09:01	231	245	SMVLFSSPPVILLIS	31.07
206	DRB1*09:01	228	242	RGSSMVLFSSPPVIL	32.28
207	DRB1*09:01	232	246	MVLFSSPPVILLISF	44.53
208	DRB1*09:01	129	143	MLGSAMSRPIIHFGS	47.82
209	DRB1*10:01	106	120	KTNMKHMAGAAAAGA	30.07
210	DRB1*10:01	104	118	KPKTNMKHMAGAAAA	31.48
211	DRB1*10:01	125	139	LGGYMLGSAMSRPII	32.36
212	DRB1*10:01	108	122	NMKHMAGAAAAGAVV	33.99
213	DRB1*10:01	124	138	GLGGYMLGSAMSRPI	34.28

214	DRB1*10:01	107	121	TNMKHMAGAAAAGAV	34.34
215	DRB1*10:01	105	119	PKTNMKHMAGAAAAG	35.27
216	DRB1*10:01	223	237	QAYYQRGSSMVLFSS	37.37
217	DRB1*10:01	230	244	SSMVLFSSPPVILLI	37.51
218	DRB1*10:01	231	245	SMVLFSSPPVILLIS	39.19
219	DRB1*10:01	229	243	GSSMVLFSSPPVILL	40.56
220	DRB1*10:01	222	236	SQAYYQRGSSMVLFS	42.71
221	DRB1*10:01	126	140	GGYMLGSAMSRPIIH	43.38
222	DRB1*10:01	127	141	GYMLGSAMSRPIIHF	48.02
223	DRB1*11:02	229	243	GSSMVLFSSPPVILL	40.89
224	DRB1*11:02	230	244	SSMVLFSSPPVILLI	47.89
225	DRB1*11:14	230	244	SSMVLFSSPPVILLI	16.54
226	DRB1*11:14	229	243	GSSMVLFSSPPVILL	17.72
227	DRB1*11:14	231	245	SMVLFSSPPVILLIS	20.32
228	DRB1*11:14	228	242	RGSSMVLFSSPPVIL	22.37
229	DRB1*11:14	232	246	MVLFSSPPVILLISF	36.97
230	DRB1*11:14	227	241	QRGSSMVLFSSPPVI	45.24
231	DRB1*11:65	229	243	GSSMVLFSSPPVILL	40.89
232	DRB1*11:65	230	244	SSMVLFSSPPVILLI	47.89
233	DRB1*13:01	229	243	GSSMVLFSSPPVILL	40.89
234	DRB1*13:01	230	244	SSMVLFSSPPVILLI	47.89
235	DRB1*13:02	230	244	SSMVLFSSPPVILLI	16.54
236	DRB1*13:02	229	243	GSSMVLFSSPPVILL	17.72
237	DRB1*13:02	231	245	SMVLFSSPPVILLIS	20.32
238	DRB1*13:02	228	242	RGSSMVLFSSPPVIL	22.37
239	DRB1*13:02	232	246	MVLFSSPPVILLISF	36.97
240	DRB1*13:02	227	241	QRGSSMVLFSSPPVI	45.24
241	DRB1*13:23	230	244	SSMVLFSSPPVILLI	16.54
242	DRB1*13:23	229	243	GSSMVLFSSPPVILL	17.72
243	DRB1*13:23	231	245	SMVLFSSPPVILLIS	20.32
244	DRB1*13:23	228	242	RGSSMVLFSSPPVIL	22.37
245	DRB1*13:23	232	246	MVLFSSPPVILLISF	36.97
246	DRB1*13:23	227	241	QRGSSMVLFSSPPVI	45.24
247	DRB1*13:96	230	244	SSMVLFSSPPVILLI	27.77
248	DRB1*13:96	229	243	GSSMVLFSSPPVILL	31.89
249	DRB1*13:96	231	245	SMVLFSSPPVILLIS	32.08
250	DRB1*13:96	228	242	RGSSMVLFSSPPVIL	45.95
251	DRB1*13:97	230	244	SSMVLFSSPPVILLI	16.54
252	DRB1*13:97	229	243	GSSMVLFSSPPVILL	17.72
253	DRB1*13:97	231	245	SMVLFSSPPVILLIS	20.32
254	DRB1*13:97	228	242	RGSSMVLFSSPPVIL	22.37
255	DRB1*13:97	232	246	MVLFSSPPVILLISF	36.97
256	DRB1*13:97	227	241	QRGSSMVLFSSPPVI	45.24
257	DRB1*14:01	230	244	SSMVLFSSPPVILLI	46.08
258	DRB1*14:01	229	243	GSSMVLFSSPPVILL	46.16
259	DRB1*14:32	229	243	GSSMVLFSSPPVILL	31.39
260	DRB1*14:32	230	244	SSMVLFSSPPVILLI	31.90
261	DRB1*14:32	231	245	SMVLFSSPPVILLIS	38.51
262	DRB1*14:32	228	242	RGSSMVLFSSPPVIL	38.61
263	DRB1*14:54	230	244	SSMVLFSSPPVILLI	46.08
264	DRB1*14:54	229	243	GSSMVLFSSPPVILL	46.16
265	DRB1*15:01	229	243	GSSMVLFSSPPVILL	7.85
266	DRB1*15:01	228	242	RGSSMVLFSSPPVIL	8.91
267	DRB1*15:01	230	244	SSMVLFSSPPVILLI	9.27
268	DRB1*15:01	227	241	QRGSSMVLFSSPPVI	11.59
269	DRB1*15:01	231	245	SMVLFSSPPVILLIS	16.16
270	DRB1*15:01	226	240	YQRGSSMVLFSSPPV	26.85
271	DRB1*15:02	229	243	GSSMVLFSSPPVILL	15.69
272	DRB1*15:02	230	244	SSMVLFSSPPVILLI	18.32
273	DRB1*15:02	228	242	RGSSMVLFSSPPVIL	19.33
274	DRB1*15:02	227	241	QRGSSMVLFSSPPVI	27.71
275	DRB1*15:02	231	245	SMVLFSSPPVILLIS	31.93
276	DRB1*15:03	229	243	GSSMVLFSSPPVILL	17.57
277	DRB1*15:03	230	244	SSMVLFSSPPVILLI	19.61
278	DRB1*15:03	228	242	RGSSMVLFSSPPVIL	21.19
279	DRB1*15:03	227	241	QRGSSMVLFSSPPVI	28.49
280	DRB1*15:03	231	245	SMVLFSSPPVILLIS	29.05
281	DRB1*15:06	229	243	GSSMVLFSSPPVILL	7.85
282	DRB1*15:06	228	242	RGSSMVLFSSPPVIL	8.91
283	DRB1*15:06	230	244	SSMVLFSSPPVILLI	9.27
284	DRB1*15:06	227	241	QRGSSMVLFSSPPVI	11.59
285	DRB1*15:06	231	245	SMVLFSSPPVILLIS	16.16
286	DRB1*15:06	226	240	YQRGSSMVLFSSPPV	26.85
287	DRB1*15:07	229	243	GSSMVLFSSPPVILL	11.44
288	DRB1*15:07	228	242	RGSSMVLFSSPPVIL	13.25
289	DRB1*15:07	230	244	SSMVLFSSPPVILLI	13.77
290	DRB1*15:07	227	241	QRGSSMVLFSSPPVI	18.01
291	DRB1*15:07	231	245	SMVLFSSPPVILLIS	23.33
292	DRB1*15:07	226	240	YQRGSSMVLFSSPPV	42.03
293	DRB1*15:15	229	243	GSSMVLFSSPPVILL	19.30
294	DRB1*15:15	228	242	RGSSMVLFSSPPVIL	22.90
295	DRB1*15:15	230	244	SSMVLFSSPPVILLI	23.51
296	DRB1*15:15	227	241	QRGSSMVLFSSPPVI	32.69
297	DRB1*15:15	231	245	SMVLFSSPPVILLIS	39.44
298	DRB1*15:37	229	243	GSSMVLFSSPPVILL	14.70
299	DRB1*15:37	228	242	RGSSMVLFSSPPVIL	17.96
300	DRB1*15:37	230	244	SSMVLFSSPPVILLI	18.02
301	DRB1*15:37	227	241	QRGSSMVLFSSPPVI	26.69
302	DRB1*15:37	231	245	SMVLFSSPPVILLIS	33.36
303	DRB1*16:01	229	243	GSSMVLFSSPPVILL	44.40
304	DRB1*16:02	229	243	GSSMVLFSSPPVILL	24.00
305	DRB1*16:02	228	242	RGSSMVLFSSPPVIL	28.05
306	DRB1*16:02	230	244	SSMVLFSSPPVILLI	31.26
307	DRB1*16:02	227	241	QRGSSMVLFSSPPVI	40.22
308	DRB1*16:02	223	237	QAYYQRGSSMVLFSS	42.04
309	DRB1*16:02	222	236	SQAYYQRGSSMVLFS	44.81
310	DRB1*16:05	229	243	GSSMVLFSSPPVILL	30.97
311	DRB1*16:05	230	244	SSMVLFSSPPVILLI	36.81
312	DRB1*16:05	228	242	RGSSMVLFSSPPVIL	41.62
313	DRB1*16:09	229	243	GSSMVLFSSPPVILL	37.42
314	DRB1*16:09	230	244	SSMVLFSSPPVILLI	45.49
315	DRB1*16:09	228	242	RGSSMVLFSSPPVIL	49.59

Table S5. Peptides (18-mer) of PRP^C binding with high affinity (IC_{50} < 50 nM) to HLA-II molecules.

Index	Allele	Start	End	Peptide	min(IC_{50})
1	DRB1*01:01	104	121	KPKTNMKHMAGAAAAGAV	40.57
2	DRB1*01:01	106	123	KTNMKHMAGAAAAGAVVG	45.40
3	DRB1*01:01	105	122	PKTNMKHMAGAAAAGAVV	41.81
4	DRB1*01:01	103	120	SKPKTNMKHMAGAAAAGA	41.32
5	DRB1*01:18	104	121	KPKTNMKHMAGAAAAGAV	44.95
6	DRB1*01:18	106	123	KTNMKHMAGAAAAGAVVG	49.85
7	DRB1*01:18	105	122	PKTNMKHMAGAAAAGAVV	45.73
8	DRB1*01:18	103	120	SKPKTNMKHMAGAAAAGA	46.74
9	DRB1*01:20	104	121	KPKTNMKHMAGAAAAGAV	43.48
10	DRB1*01:20	106	123	KTNMKHMAGAAAAGAVVG	45.77
11	DRB1*01:20	105	122	PKTNMKHMAGAAAAGAVV	43.13
12	DRB1*01:20	103	120	SKPKTNMKHMAGAAAAGA	45.02
13	DRB1*15:01	229	246	GSSMVLFSSPPVILLISF	32.76
14	DRB1*15:01	227	244	QRGSSMVLFSSPPVILLI	30.91
15	DRB1*15:01	228	245	RGSSMVLFSSPPVILLIS	31.08
16	DRB1*15:01	230	247	SSMVLFSSPPVILLISFL	45.29
17	DRB1*15:01	226	243	YQRGSSMVLFSSPPVILL	31.89
18	DRB1*15:01	225	242	YYQRGSSMVLFSSPPVIL	45.47
19	DRB1*15:02	227	244	QRGSSMVLFSSPPVILLI	49.16
20	DRB1*15:02	228	245	RGSSMVLFSSPPVILLIS	49.60
21	DRB1*15:06	229	246	GSSMVLFSSPPVILLISF	32.76
22	DRB1*15:06	227	244	QRGSSMVLFSSPPVILLI	30.91
23	DRB1*15:06	228	245	RGSSMVLFSSPPVILLIS	31.08
24	DRB1*15:06	230	247	SSMVLFSSPPVILLISFL	45.29
25	DRB1*15:06	226	243	YQRGSSMVLFSSPPVILL	31.89
26	DRB1*15:06	225	242	YYQRGSSMVLFSSPPVIL	45.47

Table S6. Peptides (22-mer) of PRP^C binding with high affinity (IC_{50} < 50 nM) to HLA-II molecules.

Index	Allele	Start	End	Peptide	min(IC_{50})
1	DRB1*01:01	120	141	AVVGGLGGYMLGSAMSRPIIHF	41.34
2	DRB1*01:01	224	245	AYYQRGSSMVLFSSPPVILLIS	44.62
3	DRB1*01:01	219	240	ERESQAYYQRGSSMVLFSSPPV	33.36
4	DRB1*01:01	221	242	ESQAYYQRGSSMVLFSSPPVIL	31.14
5	DRB1*01:01	119	140	GAVVGGLGGYMLGSAMSRPIIH	48.17
6	DRB1*01:01	123	144	GGLGGYMLGSAMSRPIIHFGSD	41.93
7	DRB1*01:01	124	145	GLGGYMLGSAMSRPIIHFGSDY	44.44
8	DRB1*01:01	104	125	KPKTNMKHMAGAAAAGAVVGGL	11.43
9	DRB1*01:01	101	122	KPSKPKTNMKHMAGAAAAGAVV	11.19
10	DRB1*01:01	106	127	KTNMKHMAGAAAAGAVVGGLGG	13.15
11	DRB1*01:01	125	146	LGGYMLGSAMSRPIIHFGSDYE	49.95
12	DRB1*01:01	100	121	NKPSKPKTNMKHMAGAAAAGAV	11.51
13	DRB1*01:01	108	129	NMKHMAGAAAAGAVVGGLGGYM	47.22
14	DRB1*01:01	105	126	PKTNMKHMAGAAAAGAVVGGLG	11.99
15	DRB1*01:01	102	123	PSKPKTNMKHMAGAAAAGAVVG	11.07
16	DRB1*01:01	223	244	QAYYQRGSSMVLFSSPPVILLI	33.97
17	DRB1*01:01	98	119	QWNKPSKPKTNMKHMAGAAAAG	16.89
18	DRB1*01:01	217	238	QYERESQAYYQRGSSMVLFSSP	34.65
19	DRB1*01:01	220	241	RESQAYYQRGSSMVLFSSPPVI	31.87
20	DRB1*01:01	103	124	SKPKTNMKHMAGAAAAGAVVGG	11.16
21	DRB1*01:01	222	243	SQAYYQRGSSMVLFSSPPVILL	31.05
22	DRB1*01:01	97	118	SQWNKPSKPKTNMKHMAGAAAA	24.66
23	DRB1*01:01	107	128	TNMKHMAGAAAAGAVVGGLGGY	23.58
24	DRB1*01:01	216	237	TQYERESQAYYQRGSSMVLFSS	36.19

25	DRB1*01:01	122	143	VGGLGGYMLGSAMSRPIIHFGS	40.95
26	DRB1*01:01	121	142	VVGGLGGYMLGSAMSRPIIHFG	40.91
27	DRB1*01:01	99	120	WNKPSKPKTNMKHMAGAAAAGA	12.11
28	DRB1*01:01	218	239	YERESQAYYQRGSSMVLFSSPP	34.31
29	DRB1*01:02	104	125	KPKTNMKHMAGAAAAGAVVGGL	41.26
30	DRB1*01:02	101	122	KPSKPKTNMKHMAGAAAAGAVV	40.60
31	DRB1*01:02	106	127	KTNMKHMAGAAAAGAVVGGLGG	48.16
32	DRB1*01:02	100	121	NKPSKPKTNMKHMAGAAAAGAV	44.16
33	DRB1*01:02	105	126	PKTNMKHMAGAAAAGAVVGGLG	43.60
34	DRB1*01:02	102	123	PSKPKTNMKHMAGAAAAGAVVG	40.00
35	DRB1*01:02	103	124	SKPKTNMKHMAGAAAAGAVVGG	40.19
36	DRB1*01:02	99	120	WNKPSKPKTNMKHMAGAAAAGA	47.85
37	DRB1*01:18	117	138	AAGAVVGGLGGYMLGSAMSRPI	47.54
38	DRB1*01:18	118	139	AGAVVGGLGGYMLGSAMSRPII	41.12
39	DRB1*01:18	120	141	AVVGGLGGYMLGSAMSRPIIHF	36.04
40	DRB1*01:18	224	245	AYYQRGSSMVLFSSPPVILLIS	30.74
41	DRB1*01:18	219	240	ERESQAYYQRGSSMVLFSSPPV	32.42
42	DRB1*01:18	221	242	ESQAYYQRGSSMVLFSSPPVIL	27.00
43	DRB1*01:18	119	140	GAVVGGLGGYMLGSAMSRPIIH	38.44
44	DRB1*01:18	123	144	GGLGGYMLGSAMSRPIIHFGSD	38.96
45	DRB1*01:18	124	145	GLGGYMLGSAMSRPIIHFGSDY	41.66
46	DRB1*01:18	229	250	GSSMVLFSSPPVILLISFLIFL	41.31
47	DRB1*01:18	104	125	KPKTNMKHMAGAAAAGAVVGGL	14.24
48	DRB1*01:18	101	122	KPSKPKTNMKHMAGAAAAGAVV	13.96
49	DRB1*01:18	106	127	KTNMKHMAGAAAAGAVVGGLGG	16.27
50	DRB1*01:18	125	146	LGGYMLGSAMSRPIIHFGSDYE	47.23
51	DRB1*01:18	100	121	NKPSKPKTNMKHMAGAAAAGAV	14.52
52	DRB1*01:18	105	126	PKTNMKHMAGAAAAGAVVGGLG	14.91
53	DRB1*01:18	102	123	PSKPKTNMKHMAGAAAAGAVVG	13.83
54	DRB1*01:18	223	244	QAYYQRGSSMVLFSSPPVILLI	26.13
55	DRB1*01:18	227	248	QRGSSMVLFSSPPVILLISFLI	36.11
56	DRB1*01:18	98	119	QWNKPSKPKTNMKHMAGAAAAG	22.26
57	DRB1*01:18	217	238	QYERESQAYYQRGSSMVLFSSP	35.05
58	DRB1*01:18	220	241	RESQAYYQRGSSMVLFSSPPVI	29.27
59	DRB1*01:18	228	249	RGSSMVLFSSPPVILLISFLIF	37.85
60	DRB1*01:18	103	124	SKPKTNMKHMAGAAAAGAVVGG	13.92
61	DRB1*01:18	222	243	SQAYYQRGSSMVLFSSPPVILL	25.74
62	DRB1*01:18	97	118	SQWNKPSKPKTNMKHMAGAAAA	30.80
63	DRB1*01:18	107	128	TNMKHMAGAAAAGAVVGGLGGY	29.25
64	DRB1*01:18	216	237	TQYERESQAYYQRGSSMVLFSS	36.58
65	DRB1*01:18	122	143	VGGLGGYMLGSAMSRPIIHFGS	37.89
66	DRB1*01:18	121	142	VVGGLGGYMLGSAMSRPIIHFG	37.31
67	DRB1*01:18	99	120	WNKPSKPKTNMKHMAGAAAAGA	15.41
68	DRB1*01:18	218	239	YERESQAYYQRGSSMVLFSSPP	34.67
69	DRB1*01:18	226	247	YQRGSSMVLFSSPPVILLISFL	34.87
70	DRB1*01:18	225	246	YYQRGSSMVLFSSPPVILLISF	32.86
71	DRB1*01:20	117	138	AAGAVVGGLGGYMLGSAMSRPI	47.77
72	DRB1*01:20	118	139	AGAVVGGLGGYMLGSAMSRPII	45.70
73	DRB1*01:20	120	141	AVVGGLGGYMLGSAMSRPIIHF	38.50
74	DRB1*01:20	224	245	AYYQRGSSMVLFSSPPVILLIS	41.77
75	DRB1*01:20	119	140	GAVVGGLGGYMLGSAMSRPIIH	42.92
76	DRB1*01:20	123	144	GGLGGYMLGSAMSRPIIHFGSD	41.96
77	DRB1*01:20	124	145	GLGGYMLGSAMSRPIIHFGSDY	44.18
78	DRB1*01:20	104	125	KPKTNMKHMAGAAAAGAVVGGL	14.27
79	DRB1*01:20	101	122	KPSKPKTNMKHMAGAAAAGAVV	14.23

80	DRB1*01:20	106	127	KTNMKHMAGAAAAGAVVGGLGG	15.99
81	DRB1*01:20	125	146	LGGYMLGSAMSRPIIHFGSDYE	48.81
82	DRB1*01:20	100	121	NKPSKPKTNMKHMAGAAAAGAV	14.73
83	DRB1*01:20	105	126	PKTNMKHMAGAAAAGAVVGGLG	14.82
84	DRB1*01:20	102	123	PSKPKTNMKHMAGAAAAGAVVG	13.99
85	DRB1*01:20	223	244	QAYYQRGSSMVLFSSPPVILLI	43.10
86	DRB1*01:20	227	248	QRGSSMVLFSSPPVILLISFLI	44.83
87	DRB1*01:20	98	119	QWNKPSKPKTNMKHMAGAAAAG	21.97
88	DRB1*01:20	228	249	RGSSMVLFSSPPVILLISFLIF	47.52
89	DRB1*01:20	103	124	SKPKTNMKHMAGAAAAGAVVGG	14.02
90	DRB1*01:20	222	243	SQAYYQRGSSMVLFSSPPVILL	45.27
91	DRB1*01:20	97	118	SQWNKPSKPKTNMKHMAGAAAA	30.52
92	DRB1*01:20	107	128	TNMKHMAGAAAAGAVVGGLGGY	27.16
93	DRB1*01:20	122	143	VGGLGGYMLGSAMSRPIIHFGS	40.90
94	DRB1*01:20	121	142	VVGGLGGYMLGSAMSRPIIHFG	39.96
95	DRB1*01:20	99	120	WNKPSKPKTNMKHMAGAAAAGA	15.77
96	DRB1*01:20	226	247	YQRGSSMVLFSSPPVILLISFL	43.10
97	DRB1*01:20	225	246	YYQRGSSMVLFSSPPVILLISF	41.87
98	DRB1*01:24	221	242	ESQAYYQRGSSMVLFSSPPVIL	48.20
99	DRB1*01:24	104	125	KPKTNMKHMAGAAAAGAVVGGL	31.39
100	DRB1*01:24	101	122	KPSKPKTNMKHMAGAAAAGAVV	29.98
101	DRB1*01:24	106	127	KTNMKHMAGAAAAGAVVGGLGG	37.76
102	DRB1*01:24	100	121	NKPSKPKTNMKHMAGAAAAGAV	31.40
103	DRB1*01:24	105	126	PKTNMKHMAGAAAAGAVVGGLG	33.54
104	DRB1*01:24	102	123	PSKPKTNMKHMAGAAAAGAVVG	29.82
105	DRB1*01:24	223	244	QAYYQRGSSMVLFSSPPVILLI	48.34
106	DRB1*01:24	103	124	SKPKTNMKHMAGAAAAGAVVGG	30.27
107	DRB1*01:24	222	243	SQAYYQRGSSMVLFSSPPVILL	46.37
108	DRB1*01:24	99	120	WNKPSKPKTNMKHMAGAAAAGA	33.68
109	DRB1*01:29	104	125	KPKTNMKHMAGAAAAGAVVGGL	32.22
110	DRB1*01:29	101	122	KPSKPKTNMKHMAGAAAAGAVV	30.89
111	DRB1*01:29	106	127	KTNMKHMAGAAAAGAVVGGLGG	38.28
112	DRB1*01:29	100	121	NKPSKPKTNMKHMAGAAAAGAV	33.05
113	DRB1*01:29	105	126	PKTNMKHMAGAAAAGAVVGGLG	34.45
114	DRB1*01:29	102	123	PSKPKTNMKHMAGAAAAGAVVG	30.52
115	DRB1*01:29	103	124	SKPKTNMKHMAGAAAAGAVVGG	30.97
116	DRB1*01:29	99	120	WNKPSKPKTNMKHMAGAAAAGA	36.40
117	DRB1*04:01	167	188	DEYSNQNNFVHDCVNITIKQHT	47.78
118	DRB1*04:01	168	189	EYSNQNNFVHDCVNITIKQHTV	48.64
119	DRB1*04:01	166	187	MDEYSNQNNFVHDCVNITIKQH	48.26
120	DRB1*04:72	167	188	DEYSNQNNFVHDCVNITIKQHT	45.08
121	DRB1*04:72	168	189	EYSNQNNFVHDCVNITIKQHTV	45.25
122	DRB1*04:72	166	187	MDEYSNQNNFVHDCVNITIKQH	45.31
123	DRB1*04:72	165	186	PMDEYSNQNNFVHDCVNITIKQ	47.27
124	DRB1*04:72	170	191	SNQNNFVHDCVNITIKQHTVTT	49.79
125	DRB1*04:72	169	190	YSNQNNFVHDCVNITIKQHTVT	46.82
126	DRB1*07:01	120	141	AVVGGLGGYMLGSAMSRPIIHF	43.75
127	DRB1*07:01	224	245	AYYQRGSSMVLFSSPPVILLIS	43.35
128	DRB1*07:01	221	242	ESQAYYQRGSSMVLFSSPPVIL	45.20
129	DRB1*07:01	123	144	GGLGGYMLGSAMSRPIIHFGSD	48.94
130	DRB1*07:01	223	244	QAYYQRGSSMVLFSSPPVILLI	41.42
131	DRB1*07:01	222	243	SQAYYQRGSSMVLFSSPPVILL	42.15
132	DRB1*07:01	122	143	VGGLGGYMLGSAMSRPIIHFGS	46.44
133	DRB1*07:01	121	142	VVGGLGGYMLGSAMSRPIIHFG	44.77
134	DRB1*07:01	225	246	YYQRGSSMVLFSSPPVILLISF	46.96
135	DRB1*09:01	120	141	AVVGGLGGYMLGSAMSRPIIHF	39.69

136	DRB1*09:01	123	144	GGLGGYMLGSAMSRPIIHFGSD	43.50
137	DRB1*09:01	124	145	GLGGYMLGSAMSRPIIHFGSDY	46.63
138	DRB1*09:01	122	143	VGGLGGYMLGSAMSRPIIHFGS	41.48
139	DRB1*09:01	121	142	VVGGLGGYMLGSAMSRPIIHFG	40.27
140	DRB1*10:01	104	125	KPKTNMKHMAGAAAAGAVVGGL	35.74
141	DRB1*10:01	101	122	KPSKPKTNMKHMAGAAAAGAVV	36.27
142	DRB1*10:01	106	127	KTNMKHMAGAAAAGAVVGGLGG	39.84
143	DRB1*10:01	100	121	NKPSKPKTNMKHMAGAAAAGAV	39.49
144	DRB1*10:01	105	126	PKTNMKHMAGAAAAGAVVGGLG	37.21
145	DRB1*10:01	102	123	PSKPKTNMKHMAGAAAAGAVVG	35.16
146	DRB1*10:01	103	124	SKPKTNMKHMAGAAAAGAVVGG	35.11
147	DRB1*10:01	99	120	WNKPSKPKTNMKHMAGAAAAGA	41.60
148	DRB1*15:01	224	245	AYYQRGSSMVLFSSPPVILLIS	14.84
149	DRB1*15:01	221	242	ESQAYYQRGSSMVLFSSPPVIL	18.97
150	DRB1*15:01	229	250	GSSMVLFSSPPVILLISFLIFL	19.47
151	DRB1*15:01	223	244	QAYYQRGSSMVLFSSPPVILLI	15.12
152	DRB1*15:01	227	248	QRGSSMVLFSSPPVILLISFLI	15.70
153	DRB1*15:01	220	241	RESQAYYQRGSSMVLFSSPPVI	28.96
154	DRB1*15:01	228	249	RGSSMVLFSSPPVILLISFLIF	16.90
155	DRB1*15:01	222	243	SQAYYQRGSSMVLFSSPPVILL	15.82
156	DRB1*15:01	230	251	SSMVLFSSPPVILLISFLIFLI	34.93
157	DRB1*15:01	226	247	YQRGSSMVLFSSPPVILLISFL	15.10
158	DRB1*15:01	225	246	YYQRGSSMVLFSSPPVILLISF	14.84
159	DRB1*15:02	224	245	AYYQRGSSMVLFSSPPVILLIS	27.59
160	DRB1*15:02	221	242	ESQAYYQRGSSMVLFSSPPVIL	35.12
161	DRB1*15:02	229	250	GSSMVLFSSPPVILLISFLIFL	36.74
162	DRB1*15:02	223	244	QAYYQRGSSMVLFSSPPVILLI	27.35
163	DRB1*15:02	227	248	QRGSSMVLFSSPPVILLISFLI	29.79
164	DRB1*15:02	228	249	RGSSMVLFSSPPVILLISFLIF	32.11
165	DRB1*15:02	222	243	SQAYYQRGSSMVLFSSPPVILL	28.26
166	DRB1*15:02	226	247	YQRGSSMVLFSSPPVILLISFL	28.60
167	DRB1*15:02	225	246	YYQRGSSMVLFSSPPVILLISF	27.92
168	DRB1*15:03	224	245	AYYQRGSSMVLFSSPPVILLIS	37.74
169	DRB1*15:03	221	242	ESQAYYQRGSSMVLFSSPPVIL	38.39
170	DRB1*15:03	229	250	GSSMVLFSSPPVILLISFLIFL	45.63
171	DRB1*15:03	223	244	QAYYQRGSSMVLFSSPPVILLI	37.90
172	DRB1*15:03	227	248	QRGSSMVLFSSPPVILLISFLI	39.50
173	DRB1*15:03	220	241	RESQAYYQRGSSMVLFSSPPVI	47.93
174	DRB1*15:03	228	249	RGSSMVLFSSPPVILLISFLIF	41.74
175	DRB1*15:03	222	243	SQAYYQRGSSMVLFSSPPVILL	38.19
176	DRB1*15:03	226	247	YQRGSSMVLFSSPPVILLISFL	38.60
177	DRB1*15:03	225	246	YYQRGSSMVLFSSPPVILLISF	38.05
178	DRB1*15:06	224	245	AYYQRGSSMVLFSSPPVILLIS	14.84
179	DRB1*15:06	221	242	ESQAYYQRGSSMVLFSSPPVIL	18.97
180	DRB1*15:06	229	250	GSSMVLFSSPPVILLISFLIFL	19.47
181	DRB1*15:06	223	244	QAYYQRGSSMVLFSSPPVILLI	15.12
182	DRB1*15:06	227	248	QRGSSMVLFSSPPVILLISFLI	15.70
183	DRB1*15:06	220	241	RESQAYYQRGSSMVLFSSPPVI	28.96
184	DRB1*15:06	228	249	RGSSMVLFSSPPVILLISFLIF	16.90
185	DRB1*15:06	222	243	SQAYYQRGSSMVLFSSPPVILL	15.82
186	DRB1*15:06	230	251	SSMVLFSSPPVILLISFLIFLI	34.93
187	DRB1*15:06	226	247	YQRGSSMVLFSSPPVILLISFL	15.10
188	DRB1*15:06	225	246	YYQRGSSMVLFSSPPVILLISF	14.84
189	DRB1*15:07	224	245	AYYQRGSSMVLFSSPPVILLIS	23.80
190	DRB1*15:07	221	242	ESQAYYQRGSSMVLFSSPPVIL	29.09
191	DRB1*15:07	229	250	GSSMVLFSSPPVILLISFLIFL	31.69

192	DRB1*15:07	223	244	QAYYQRGSSMVLFSSPPVILLI	24.10
193	DRB1*15:07	227	248	QRGSSMVLFSSPPVILLISFLI	25.66
194	DRB1*15:07	220	241	RESQAYYQRGSSMVLFSSPPVI	42.37
195	DRB1*15:07	228	249	RGSSMVLFSSPPVILLISFLIF	27.71
196	DRB1*15:07	222	243	SQAYYQRGSSMVLFSSPPVILL	25.16
197	DRB1*15:07	226	247	YQRGSSMVLFSSPPVILLISFL	24.57
198	DRB1*15:07	225	246	YYQRGSSMVLFSSPPVILLISF	24.06
199	DRB1*15:15	224	245	AYYQRGSSMVLFSSPPVILLIS	45.18
200	DRB1*15:15	221	242	ESQAYYQRGSSMVLFSSPPVIL	44.17
201	DRB1*15:15	223	244	QAYYQRGSSMVLFSSPPVILLI	42.55
202	DRB1*15:15	222	243	SQAYYQRGSSMVLFSSPPVILL	41.70
203	DRB1*15:15	226	247	YQRGSSMVLFSSPPVILLISFL	48.40
204	DRB1*15:15	225	246	YYQRGSSMVLFSSPPVILLISF	46.89
205	DRB1*15:37	224	245	AYYQRGSSMVLFSSPPVILLIS	27.30
206	DRB1*15:37	221	242	ESQAYYQRGSSMVLFSSPPVIL	38.22
207	DRB1*15:37	229	250	GSSMVLFSSPPVILLISFLIFL	38.22
208	DRB1*15:37	223	244	QAYYQRGSSMVLFSSPPVILLI	27.71
209	DRB1*15:37	227	248	QRGSSMVLFSSPPVILLISFLI	29.97
210	DRB1*15:37	228	249	RGSSMVLFSSPPVILLISFLIF	32.76
211	DRB1*15:37	222	243	SQAYYQRGSSMVLFSSPPVILL	29.46
212	DRB1*15:37	226	247	YQRGSSMVLFSSPPVILLISFL	28.48
213	DRB1*15:37	225	246	YYQRGSSMVLFSSPPVILLISF	27.75
214	DRB1*16:02	223	244	QAYYQRGSSMVLFSSPPVILLI	47.36
215	DRB1*16:02	222	243	SQAYYQRGSSMVLFSSPPVILL	46.94
